# *Akkermansia muciniphila*: The state of the art, 18 years after its first discovery

**DOI:** 10.3389/fgstr.2022.1024393

**Published:** 2022-10-25

**Authors:** Rim Iwaza, Reham Magdy Wasfy, Grégory Dubourg, Didier Raoult, Jean-Christophe Lagier

**Affiliations:** ^1^Aix Marseille Univ, Institut de recherche pour le développement (IRD), Assistance Publique - Hôpitaux de Marseille (AP-HM), Microbes, Evolution, Phylogénie et Infection (MEPHI), Marseille, France; ^2^Institut hospitalo-universitaire (IHU)-Méditerranée Infection, Marseille, France

**Keywords:** human health, metabolic diseases, cancer, culture, probiotic, microbiota, *Akkermansia muciniphila*

## Abstract

*Akkermansia muciniphila (A. muciniphila)* is an anaerobic, Gram negative and mucin-degrading bacterium of the phylum Verrucomicrobia isolated in 2004 from human feces. Although it is a common resident in the human intestinal tract, it has also been detected in other anatomical sites. Genomic studies have revealed that *A. muciniphila* can be divided into different phylogroups with distinct metabolic properties. There is growing evidence regarding its beneficial impact on human health. Indeed, *A. muciniphila* is considered as a promising next-generation probiotic for treating cancer and metabolic disorders. The large-scale production of *A. muciniphila* is, therefore, a challenge. Beside mucin-based medium, other culture strategies have enabled its isolation. The administration of both live and pasteurized forms of *A. muciniphila* has shown to be promising in animal models. Alternatively, the administration of various prebiotics has also been assessed for enhancing its abundance in the human gut. Future prospects include human clinical trials, some of which are currently ongoing. This paper provides an overview of what is currently known about *A. muciniphila’s* phenotypical and genotypic traits, as well as its culture techniques and its connections to a number of human diseases and its potential application as an effective next generation probiotic.

## Introduction

Within the human microbiome, the gut microbiota has, to date, been the most characterized, and its function and importance in maintaining the balance between human health and pathology has been widely investigated. Alteration of the composition of the gut microbiota has been linked to several diseases, including inflammatory bowel syndrome (1), type 2 diabetes (2), and cancer (3) as well as eating disorders (4) and psychological disorders (5). Different phyla are reported in the gut, the two phyla Firmicutes and Bacteroidetes represent 90% of gut microbiota. Other reported phyla include Actinobacteria, Proteobacteria, Fusobacteria, and Verrucomicrobia (6). *A. muciniphila* is the only species in the Verrucomicrobia phylum that has been reported in the gastro-intestinal tract. Discovered and isolated from the stool of a healthy individual in 2004 by Derrien et al. (7), *A. muciniphila* relies on mucin for carbon, nitrogen and energy. Since then, it has been reported that it constitutes between 1% and 3% of the fecal microbiota and is present in more than 90% of healthy adults tested, but decreases in the elderly (8, 9). The majority of the research studies reporting the presence of *A. muciniphila* presence in the human digestive tract are based on metagenomic analysis, but only few studies have reported its isolation. The capacity of *A. muciniphila* to degrade and use mucin as a unique source of carbon and nitrogen gives it significant importance in the human gastro-intestinal tract, giving the opportunity to other bacteria to survive and grow by using the metabolites resulting from mucin degradation. These metabolites also play a role in the inflammatory status of the host (10). *A. muciniphila* was found to regulate the immune system, improve the gut barrier function and ameliorate metabolism in the case of obesity and diabetes, especially *in vitro* or in mice models (11–13). Furthermore, an association was found between high relative abundance of *A. muciniphila* and a lower incidence of obesity (14). Its abundance is found to decrease in different kind of diseases such as cancer (15–17), type 2 diabetes (18), inflammatory diseases (19) and liver diseases (20). These findings allowed the association between the presence of *A. muciniphila* and the healthy status of human beings, given that its abundance significantly decreases in many diseases. For this reason, it could be used as a marker of certain diseases with differing severity. Due to its beneficial effects on the human body, recent studies have also promoted its use as a probiotic (21, 22). To date, there are three validly published studies that reported the safety of use and the beneficial role of *A. muciniphila* in obese humans as a probiotic (12, 23, 24), while two clinical trials are in progress to evaluate the effects of the use of *A. muciniphila* in obese patients with type 2 diabetes and in hyperglycemic adults (NCT04797442/NCT05114018). The purpose of this review is to summarize what is currently known about *A. muciniphila* in terms of both the phenotypical and the genotypical characteristics, as well as its culture methods. We will also discuss its relationships with many human diseases. And most importantly, we will discuss the already established human trials and those that are still in progress focusing on and its possible use as a promising probiotic.

## The *Akkermansia* genus

Since its discovery by Derrien et al. (7) in 2004, the *Akkermansia* genus, which is a part of the division Verrucomicrobia contains only two known species: *A. muciniphila* and *A. glycaniphila* (25). However, a recent study analyzing metagenome-assembled genomes of *Akkermansia* suggested the presence of two more putative species (26), while another study cited the presence of eight different species in the genus *Akkermansia* (27). *Akkermansia* spp. are Gram-negative, non-motile, non-spore forming and anaerobic bacteria. Cells are oval shaped with a mean diameter of 0.6–1.0 μm (7).

### Taxonomy

A study analyzed 23 whole genome sequences of the *Akkermansia* genus and revealed that these strains formed four clades, divided into four species based on dDDH values (28), while a more recent study has divided *Akkermansia* strains into five distinct group (29). Moreover, it has been shown that single nucleotide polymorphisms (SNPs) were not evenly distributed throughout the *A. muciniphila* genomes, while genes in regions with high SNPs are found to be related to metabolism and cell wall/membrane envelope biogenesis (28).

When it comes to *A. muciniphila*, many genomic studies have been conducted in order to study its genomic diversity. *A. muciniphila* can be subdivided into three phylogroups, with high nucleotide diversity and distinct metabolic and functional profiles (30). However, a recent study has reported the presence of four different *Akkermansia* phylogroups, based on pangenome analysis (31). Another study analyzed different *A. muciniphila* strains from different phylogroups and revealed that each phylogroup has some specific phenotypes such as oxygen tolerance or sulfur assimilation. These phenotypes can influence the colonization of the gastrointestinal tract (32).

### Metabolic characteristics

This genus uses mucin as its only carbon and nitrogen source, but it has been proven that it can grow in a medium containing glucose, N-acetylglucosamine and N-acetylgalactosamine, when provided with other protein sources (7, 25). The uptake of these sugars can also be enhanced by adding mucin, revealing the role of other mucin-derived components in its growth (33).

*A. muciniphila* has numerous candidate mucinase-encoding genes but surprisingly lacks genes coding for canonical mucus-binding domains (27). This capacity to degrade mucin might be essential to the survival of other bacteria in the human gut, as mucin degradation by *A muciniphila* provides metabolites that supports the growth of other bacteria such as *Anaerostipes caccae* by changing the transcriptional profile to induce an increase in the expression of mucin degradation genes and a reduction in the expression of ribosomal genes (34). Among the various studies aiming to identify the enzymes involved in mucin degradation, one has succeeded in identifying a novel phospholipid‐regulated β‐galactosidase involved in mucin degradation (35). Further work revealed other beta galactosidases involved in the complex mucin degradation machinery (36). *A. muciniphila* can survive without the addition of vitamins to the medium. It was even proven in a recent study that some *A. muciniphila* strains were able to synthesize vitamin B12 (31).

### Resistance to antimicrobial agents

*A. muciniphila* and *A.glycaniphila* strains have been shown to be resistant to ampicillin and vancomycin (7, 25, 37, 38). Specifically, *A. muciniphila* Muc^T^ was also found to be resistant to other antibiotics, including metronidazole and penicillin G, but susceptible to doxycycline, imipenem, and piperacillin/tazobactam (38). This antibiotic profile can change from one strain to another. For example, another *A. muciniphila* strain isolated in 2017 was sensitive to penicillin, imipenem, ceftriaxone and amoxicillin but resistant to ofloxacin (37). In 2015, a study aimed at assembling the genome of a strain sequenced directly from a human stool sample detected its presence, by performing an in-silico prediction of eight beta lactamase genes. Moreover, three macrolide resistance genes were detected with only one sharing 65% similarity with a known macrolide gene. Finally, resistance to vancomycin, chloramphenicol, sulfonamide, tetracycline and trimethoprim was associated with only one gene (39).

## *A. muciniphila* distribution within the human body

### Digestive tract

*A. muciniphila* is a common bacterial component of the human intestinal tract (9). A study by Collado et al. found that the presence of *A. muciniphila* presence increases from 16% of the samples of one-month-old infants to 90% at 12 months, while it is present in all the adult samples. Similarly, levels also increase in early life to reach levels similar to that observed in adults within a year. On the other hand, this level decreases significantly in the elderly (8).

**Figure 1:**
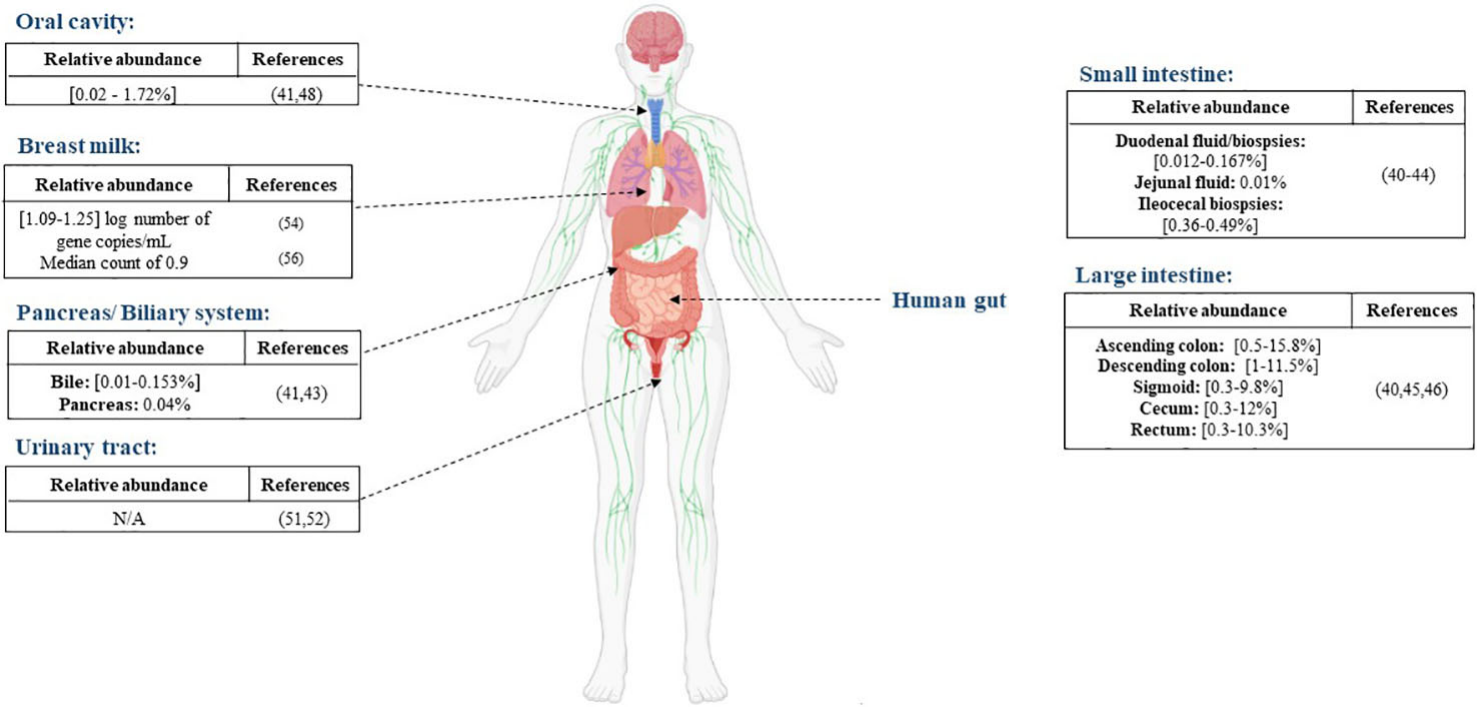
Distribution of *A. muciniphila* in different anatomical sites, the reported abundance in each site and the corresponding references.

Aiming to characterize the whole gut microbiota, Mailhe et al. collected samples from various parts of the digestive tract: the stomach, duodenum, ileum, and the left and right colon and analyzed those samples using culturomics and metagenomics. They succeeded in cultivating *Akkermansia muciniphila* in the left colon. In terms of metagenomic analysis, the Verrucomicrobia phylum, represented by the *Akkermansia* genus, was detected in the duodenum, ileum, and the right and left colon (40). Moreover, Ye et al., detected *Akkermansia*-like sequences in three out of six duodenal fluid samples (41). Another study exploring the duodenal and rectal microbiota in luminal contents and biopsy tissues in healthy volunteers found Verrucomicrobia sequences in duodenal biopsies, mucus and rectal biopsies (42). The presence of *Akkermansia* sequences was reported in the jejunal fluid, the pancreas and the bile with mean relative abundance of 0.01%, 0.05% and 0.01%, respectively, in a study exploring disturbances in the microbiome in patients undergoing pancreaticoduodenectomy (43). Analysis based on 16S rRNA genes uncovered the presence of *Akkermansia* sequences in ileocecal biopsies of patients with primary sclerosing cholangitis (PSC), ulcerative colitis and in non-inflammatory controls, with no significant differences between the different groups (44). The presence of Verrucomicrobia or *Akkermansia*-like sequences were detected much more frequently in the large intestine (45, 46) (**Figure 1**).

### Oral cavity

There is no significant evidence of the presence of *A. muciniphila* in the oral cavity. Le et al. highlighted an absence of *A. muciniphila* in the oral cavities of 47 pediatric patients after PCR screening (47). A study performed in 2017 by Coretti et al. assessed the subgingival microbiota of smokers and non-smokers with chronic periodontitis compared to a control group. They found that the Verrucomicrobia phylum was significantly lower in people with chronic periodontitis (48). *A. muciniphila* was also detected in the saliva sample of a choledocholithiasis patient. He was the only positive patient out of six and the relative abundance was very low (41) (**Figure 1**). While its presence is not abundant in the oral cavity, *A. muciniphila* can be a potential therapeutic agent for periodontitis. In an experimental periodontitis mouse model, *A. muciniphila* showed protective effects by decreasing inflammatory cell infiltration and reducing alveolar bone loss (49).

### Urinary tract

Although urine was long considered sterile, some studies have proved the presence of resident microorganisms by using culture and molecular based techniques (50). Few studies have detected the presence of *A. muciniphila* in the urine. Mansour et al. analyzed tissue and urine samples from patients with bladder carcinoma in order to compare the microbiota in both type of samples. Sequencing results showed that the *Akkermansia* genus was present in both type of samples but was over-represented in the tissue samples compared to the urine samples (51). Another sequencing-based study reported a decrease in the levels of the phylum Verrucomicrobia in urine samples from an elderly Type 2-diabetes mellitus group compared with a control group (52).

### Human breast milk

Human milk contains nutrients providing immunological and other health benefits to new-born babies. Studies on human milk show that it provides a source of commensal microorganisms for the new-born gut (53). As for *A. muciniphila*, its presence in human breast milk was reported for the first time in a study conducted by Collado et al. (54) The study showed that *A. muciniphila* was found in milk samples taken from women shortly after giving birth (colostrum), as well as at one and six months, with mean concentrations of 1.25, 1.09, and 1.20 log number of gene copies/mL, respectively. Moreover, they demonstrated that *A. muciniphila* was more abundant in overweight mothers than in normal weight mothers. Another study in 2014 discovered the presence of *Akkermansia*-like species using 16S rRNA sequencing in human breast tissue samples of 43 women (aged 18 to 90 years) (55). In addition, *A. muciniphila* was also found by qPCR analysis in milk colostrum samples collected from 11 women after an elective caesarean section, with a median count of 0.9 (56). Furthermore, metagenomic analysis of breast milk samples from healthy Korean mothers detected the presence of the *Akkermansia* genus (57). Finally, in a study aiming to evaluate the impact of maternal breast milk composition on children who develop coeliac disease (CD), milk samples were collected from mothers with a genetic predisposition to CD and a control group. The genus *Akkermansia* was found in milk from mothers in the CD group and the control group but was more abundant in the CD group (58). The presence of *A. muciniphila* in the human breast milk might be due to its ability to use human milk oligosaccharides (59). Although it is important to note that this ability is strain dependent (60).

## *A. muciniphila* culture methods

The *Akkermansia* genus was isolated for the first time from a human stool sample, with *A. muciniphila* being the type species using a basal medium supplemented with 0.25% gastric mucin and 0.7% rumen fluid. The human stool was serially diluted into sterile anaerobic Ringer’s solution containing 0.5 g cysteine. Each dilution was inoculated in the medium as described previously. Pure colonies were isolated using the same medium containing 0.75% agar. Since then, other studies have used the same medium in order to isolate other *Akkermansia* strains (61). Twelve years later, the same medium enabled the cultivation, from reticulated 193 python faeces (25) of *A. glycaniphila*, which depends on mucin as its only energy source for carbon and nitrogen. In a recent study focusing on distinguishing the fast and slow growing bacteria of the faecal microbiota by changing the dilution rates in mucin-supplemented media, *A. muciniphila* was isolated in low dilution rates (62). It was also suggested that the growth of *A. muciniphila* is promoted in a media rich in sugar and mucin (63). To understand how *A. muciniphila* adapts to mucin, transcriptomic and metabolomic analysis showed an upregulation of genes related to energy metabolism and cell growth in the presence of 0.5% of mucin, correlated with smaller diameter of the cells, a sign of bacterial division, and proliferation. Moreover, enzymes such as fucosidase, beta-galactosidase and hexosaminidase were also overexpressed to degrade mucins into oligosaccharides and eventually monosaccharides to use them as a source of energy (64). Another study comparing the growth of *A. muciniphila* in static and dynamic culture simulating the physiological conditions in the colon showed that the biomass of *A.muciniphila* in dynamic culture was significantly higher after 48 hours compared to under static conditions. The same study tested the growth of *A. muciniphila* in five different culture conditions: human mucin, porcine mucin, brain heart infusion (BHI) medium only, or BHI supplemented with porcine mucin or human mucin. *A muciniphila* can grow in all the media tested, but the lowest biomass was found in BHI only, and human mucin is the most ideal for the cell growth (65).

However, some studies have proved that *A. muciniphila* can be isolated without mucin-based media culture, such as from a blood culture sample after 72 hours of subculture on Columbia agar with 5% sheep blood (37). Similarly, another strain was isolated from a stool sample after diluting it in pre-reduced phosphate-buffered saline (PBS), plating on Columbia blood agar supplemented with 5% horse blood, and subjected to two to four days of incubation at 37°C under an H_2_-CO_2_-N_2_ (1:1:8 [vol/vol/vol]) gas mixture (66). Finally, culturomics techniques enabled the isolation of *A. muciniphila* from fresh stool samples using the following anaerobic culture conditions at 37 °C: culture bottle containing 5% sheep blood and 5% rumen fluid, YCFA medium, YCFA solid medium, reinforced clostridiales solid medium, brain heart infusion (BHI) solid medium, Columbia solid medium and, finally, De Man, Rogosa and Sharpe (MRS) solid medium (67, 68) (**Table 1**).

**Table 1:** *A. muciniphila* culture methods.

Strain	Sample	Medium used/Culture conditions	Authors	Year
***A. muciniphila***	Stool sample	0.4 g KH_2_PO_4_; 0.53 g Na_2_HPO_4_; 0.3 g NaCl; 4 g NaHCO_3_; 0.3 g NH_4_Cl; 0.25 g Na_2_S.7–9H_2_O; 0.1 g MgCl_2_.6H_2_O; 0.11 g CaCl_2_; 1 ml alkaline trace element solution; 1 ml acid trace element solution; 0.5 mg resazurin and 1 ml vitamin solution 0.25% gastric mucin and 0.7% rumen fluid.	Derrien et al. (7)	2004
	Blood culture sample	Incubation of blood culture sample for 96 hours Colonies isolated after 72 hours of subculture on Columbia agar with 5% sheep blood	Dubourg et al. (37)	2017
	Fecal sample	Mucin-supplemented media, low dilution rates	Adamberg et al. (62)	2018
	Intestinal microbiota samples	Bacterial growth media (containing sugars, nitrogen, vitamins, minerals, hematin, amino acids and mucin)	Yousi et al. (63)	2019
	Stool samples	Culturomics in anaerobic conditions with the following media: Culture bottle with 5% sheep blood and 5% rumen fluid/YCFA liquid medium and solid/Reinforced clostridiales solid/Brain heart infusion solid/Colombia solid/De Man, Rogosa and Sharpe solid at 37°C	Lagier et al. (67) Diakite et al. (68)	2016/2020
	Stool sample	Columbia blood agar supplemented with 5% horse blood and two to four days of incubation at 37 °C under a H_2_-CO_2_-N_2_ (1:1:8 [vol/vol/vol]) gas mixture	Ogata et al. (66)	2020

Table resuming the different studies that have succeeded in cultivating A. muciniphila, the year of publication, the origin of the sample and the media and culture conditions used in each study.

The growth of *A. muciniphila* has been proven to be pH dependent. The optimum pH was 6.5. Low pH strongly inhibits its growth, explaining its abundance in the distal colon in comparison to the proximal colon (69, 70). *A. muciniphila* also showed high tolerance to oxygen (up to 72 hours) (71). When oxygen is present at nanomolar concentrations, its growth rate and yield were increased compared to those observed in strict anaerobic conditions. This is due to the presence of cytochrome bd complex that can function as a terminal oxidase (72). *A. muciniphila* showed high tolerance to different temperatures (4°C, 22°C, and 37°C). In contrast, cell viability showed significant decrease at 44°C. In this study, its stability and tolerance to the different gastrointestinal conditions were evaluated. Interestingly, *A. muciniphila* showed stability after exposure to simulated gastrointestinal conditions. Other evaluations might be needed in order to understand the effect of stress on the metabolism and the adhesion properties of the bacterium (71).

Other studies have tested different growth conditions for *A. muciniphila*. For example, as mentioned before, a study proved that *A. muciniphila* is also able to grow on human milk *in vitro* and degrade its oligosaccharides, which is explained by proteomic analysis showing an increase in the expression of glycan degrading enzymes such as α-L-fucosidases, β-galactosidases, exo-α-sialidases and β-acetylhexosaminidases (59). *A. muciniphila* does not code for the enzyme that mediates the conversion of fructose-6-phosphate (Fru6P) to glucosamine-6-phosphate (GlcN6P), which is essential in peptidoglycan formation. This finding suggests that N-acetylglucosamine found in mucin is crucial for the growth of *A. muciniphila*, thus explaining its importance and the adaptation of *A. muciniphila* to its components (73). In contrast, bile salts were found to impede the growth of *A. muciniphila*, except for sodium deoxycholate which increased its growth (74).

## *A. muciniphila* and health

### Cancer

The association between cancer and changes in the gut microbiota in humans has been widely investigated. More specifically, the role of *A. muciniphila* in different types of cancer has been assessed (75). One study highlighted the decrease in the abundance of faecal *A. muciniphila* among non-small-cell lung cancer patients compared to controls (15), through metagenomic and metabolomic profiling, while an increase was detected using real time PCR on gut mucosal tissues samples of colorectal cancer patients compared to controls (16), Similarly, an abundance of *A. muciniphila* along with other bacteria was significantly increased in patients with different gastrointestinal cancer such as esophageal, gastric and colorectal cancer, compared to the control group (17) (**Table 2**).

**Table 2:** Association between *A. muciniphila* and different clinical diseases.

Type of diseases	Pathology	Samples	Cohort	Technique	Abundance of *A. muciniphila*	Other findings	References
**Metabolic disorders**	**Acute appendicitis**	appendices, cecal biopsies and faecal samples	70 patients with appendicitis/400 controls	rRNA-based FISH	↓	*A. muciniphila is* inversely related to the severity of the disease.	(76)
	**Inflammatory bowel disease (IBD)**	Biopsies	46 IBD/20 controls	Real-time PCR	↓	*x*	(77)
	**Binge eating disorder (BED)**	Stool samples	101 obese patients with/without BED	Sequencing and subsequent bioinformatics	↓	x	(78)
	**Ulcerative colitis (UC)**	Colonic biopsies and mucus brushings	20 patients with active UC/14 with quiescent UC/20 healthy controls	Real-time PCR	↓	Inverse relationship between *A. muciniphila* and inflammation	(19)
	**Alcoholic liver disease (ALD)**	Fecal samples	21 patients with ALD/16 non-obese healthy controls	Quantitative PCR	↓	Decrease of faecal *A. muciniphila* indirectly correlated with hepatic disease severity	(20)
	**Obesity**	Fecal samples	164 participants with variable geographical origin, diet, age, and gender	Metagenomics	↓	Fecal salinity was associated with obesity and a depletion in anti-obesity *A. muciniphila*	(79)
			21 adult women with severe or moderate obesity	Metagenomics/Quantitative PCR	↓	Significant lower *A. muciniphila* abundance in severe obesity than in moderate obesity	(80)
			20 overweight children/20 control children	Quantitative PCR	↓	*x*	(81)
			17 lean/15 obese females		↓	*x*	(82)
	**Type 2 diabetes (T2D)**	Fecal samples	134 Danish adults with prediabetes/134 controls	Sequencing	↓	x	(83)
			182 lean/obese individuals with T2D	Metagenomic/Metabolomics	↓	Significant decrease of *A. muciniphila* abundance in lean individuals with T2D than without T2D, but not in the comparison of obese individuals with and without T2D.	(18)
			345 patients with T2D/nondiabetic controls	Sequencing	↑	x	(84)
		Urine samples	70 female T2DM patients/70 healthy females		↓	Decreased Akkermansia muciniphila was associated with high Fasting blood glucose and urine glucose	(85)
	**Clostridioides difficile infection (CDI)**	Fecal samples	50 CDI patients/50 healthy controls	Real-time Quantitative PCR	↑	x	(86)
**Cancer**	**Non-small cell lung cancer (NSCLC)**	Stool samples	11 NSCLC patients/8 controls	Metagenomics/Metabolomics	↓	x	(15)
	**colorectal cancer (CRC)**	gut mucosal tissues	18 CRC patients/18 non-CRC controls	Quantitative PCR	↑	x	(16)
	**Gastrointestinal cancer**	Stool samples	130 gastrointestinal cancer patients/147 healthy controls	16S rRNA sequencing	↑	x	(17)
**Other diseases**	** Allergic asthma**	stool samples	92 children (between 3 and 8) with asthma/88 healthy children	Quantitative PCR	↓	x	(87)
	**Atopic dermatitis (AD)/Food allergy**	Fecal samples	82 children with AD with absence and presence of food allergy	16S rRNA microbial analysis	↑	Fecal microbiome of children with AD and food allergy harbored relatively more *A. muciniphila* than children with AD without food allergy	(88)
	**Psoriasis**	Fecal samples	14 psoriasis patients/14 healthy controls	16S rDNA sequencing	↓	x	(89)
	**CaOx dihydrate (COD) and monohydrate (COM) lithiasis**	Fecal samples	24 patients diagnosed with CaOx lithiasis	Real-time PCR	↓	x	(90)
	**Autism spectrum disorder (ASD)**	Fecal samples	23 children with ASD/22 typically developing siblings/9 unrelated community controls	Real-time Quantitative PCR	↓	x	(91)

Table resuming the different studies that associated *A. muciniphila* with different diseases, the cohort, type of sample, and technique used in each study, as well as the change in the abundance of *A. muciniphila* and the references. ↑: Increase in abundance, ↓: Decrease in abundance.

A study performed on pancreatic cancer xenograft mice model showed an increase in *A. muciniphila* in the guts of mice receiving Gemcitabine treatment, as well as a decrease in tumour volume (92). In a prostate cancer mice model, the relative abundance of *A. muciniphila* in the gut was decreased. However, this decrease was reversed after receiving androgen deprivation therapy (93).

In other studies concentrating on the role of gut microbiota in the response to anti-PD1 (Programmed cell Death protein 1) immunotherapy, the presence of species such as *Bifidobacterium breve*, *Bifidobacterium longum*, *Faecalibacterium prausnitzii* and, most importantly, *A. muciniphila* in the gastro-intestinal tract of cancer patients was associated with a stronger immune response to the therapy and subsequently an extended survival of these patients (94). In another study based on anti-PD1 therapy for non-small cell lung cancer (NSCLC), two genera, *Akkermansia* and *Olsenella*, were significantly higher in the stable disease group than in the progressive disease group (95). Similarly, gastric cancer patients showed an enrichment for the genus *Akkermansia* before and after radical distal gastrectomy (96).

In epithelial tumours, metagenomic analysis of stool samples from patients receiving immune checkpoint inhibitors showed correlations between clinical responses to the treatment and the relative abundance of *A. muciniphila* (97). The same team also found an increase in *A. muciniphila* levels in patients responding favourably to immune checkpoint blockade treatment in a cohort of renal cell carcinoma patients (98).

In a study of anti-colon cancer therapy based on treatment with FOLFOX, it was demonstrated that the abundance of *A. muciniphila* significantly increased in patients receiving the treatment, which was positively correlated with the therapeutic effect (99).

In terms of colorectal cancer (CRC), it has been demonstrated that CRC tissues increase the expression of mucin2 compared to normal mucosa (100).

Finally, in a randomized trial evaluating the impact of probiotic supplementation on the outcome of gut microbiome and metastatic renal cell carcinoma (mRCC), patients who had received a treatment and had been supplemented with probiotics present a higher abundance of *A. muciniphila* in the gut (101). Furthermore, there was a positive and significant association between the presence of *A. muciniphila* and the clinical benefit of the treatment (101).

### Metabolic diseases

The abundance of *A. muciniphila* is decreased in many metabolic disorders, such as inflammatory bowel diseases, appendicitis and obesity (76, 77, 79) suggesting its association with healthy intestine and normal mucosa. Eating disorders, such as binge eating disorder (78) (**Table 2**) have also been associated with a decrease in the levels of *A. muciniphila*.

These findings reveal the importance of *A. muciniphila* as a biomarker of health status (102). Many studies targeted treating metabolic diseases have focused on tracking the levels of *A. muciniphila* to assess the success of the therapy (103).

#### Liver diseases

Liver diseases are associated with changes in the gut microbiota, specifically a decrease in the levels of *A. muciniphila*. Grander et al. suggested that the decrease in levels of *A. muciniphila* in alcoholic liver disease is indirectly correlated with disease severity (20) (**Table 2**). In contrast, other studies have highlighted an increase in *A. muciniphila* after treatment. For example, in non-alcoholic liver disease mice models, it was reported that treatment with Bilberry anthocyanins increases the levels of *A. muciniphila* in the digestive tract, associated with the efficacy of the treatment on NAFLD (104). Similarly, another study using an alcoholic liver disease mice model showed that treatment with berberine also cause an increase in the levels of *A. muciniphila* (105).

#### Obesity

*A. muciniphila* levels are negatively correlated with obesity. Studies have shown that the abundance of *A. muciniphila* decreases significantly in overweight/obese preschool children (81), and in obese adult women (82) compared to the normal weight/lean group. Moreover, its abundance is even lower in severe obesity (80) (**Table 2**). The presence of *A. muciniphila* is also associated with the normal weight gain in pregnant women (106). The beneficial effects of *A. muciniphila* can also be observed in obese adults after a six-week calorie restriction period followed by a six-week weight stabilization diet. The adults included in this study had a healthier metabolic status when the abundance of *A. muciniphila* was high. Moreover, *A. muciniphila* was associated with other microbial species related to health (107).

However, another study on obese patients undergoing bariatric surgery, gastric banding or the Roux-en-Y gastric bypass procedure showed that the relative abundance of *A. muciniphila* was inversely correlated with the severity of obesity but was not associated with glucose homeostasis markers. Furthermore, a significant increase in the relative abundance of *A. muciniphila* was observed after the Roux-en-Y gastric bypass procedure but was not correlated with metabolic improvement (80).

When it comes to the mechanism of *A. muciniphila* in controlling obesity, evidence have shown that *A. muciniphila* stimulates glucagon-like peptide-1 (GLP-1) production by intestinal cells, leading overall to an improvement in insulin sensitivity, glucose tolerance and suppressing appetite (108).

#### Diabetes

In relation to diabetes, some studies have provided evidence revealing the association between *A. muciniphila* and the metabolism of glucose and its dysregulation. Allin et al. showed that abundance of *A. muciniphila* is decreased in individuals with prediabetes (83). One study showed that in lean individuals with T2D, the levels of *A. muciniphila* are lower compared to the control group, which is not the case with obese T2D patients (18). Another study also showed a decrease in *A. muciniphila* in T2D patients, associated with higher fasting blood glucose and urine glucose (85). However, one metagenomic study on a Chinese population found that some of the genes in *A. muciniphila* were enriched in type 2 diabetic subjects, perhaps due to differences in genes and lifestyle (84) (**Table 2**). In type 1 diabetes (T1D), NGS analysis of stool samples from T1D patients receiving probiotics showed an elevation of *Bifidobacterium animalis*, *A. muciniphila* and *Lactobacillus salivarius* associated with reduced fasting blood glucose levels and improvement of glycated hemoglobin levels (109). Plovier et al. recently highlighted the effect of pasteurized *A. muciniphila* to diminish fat mass development, insulin resistance, and dyslipidemia in mice. They also demonstrated that the outer membrane protein Amuc 1100 is involved in the bacterial-to-host contact through Toll-like receptor 2 signaling. Moreover, this protein partially mimics the effects of *A. muciniphila* on insulin resistance and gut barrier modification (12).

#### Inflammatory bowel diseases

Earley et al. quantified *A. muciniphila* in colonic biopsies and mucous swabs from patients with active ulcerative colitis and quiescent ulcerative colitis. They demonstrated that patients with active ulcerative colitis had a reduced abundance of *A. muciniphila* compared to quiescent ulcerative colitis and controls (19). Studies focusing on inflammatory bowel disease have shown that mucolytic bacteria levels increase in IBD patients. However levels of *A. muciniphila* reduce, mainly due to the potential anti-inflammatory role of *A. muciniphila* (77). Another observational study has suggested that the relative abundance of *A. muciniphila* is inversely correlated to pain reduction in a cohort of IBS patients (110).

#### Other diseases

The depletion of *A. muciniphila* has also been associated with several allergic disorders, suggesting a potential educational role toward immunity. For example, decreased levels of *A. muciniphila* and *Faecalibacterium prausnitzii* in stool samples of patients with allergic asthma have been reported (87). In children with atopic dermatitis (AD), the presence of a microbial signature made it possible to differentiate between the presence and absence of food allergies. The fecal microbiome of children with AD and food allergies contains relatively less *B. breve*, *B. adolescentis*, *F. prausnitzii*, and *A. muciniphila* and more *E. coli* and *B. pseudocatenulatum* than children with AD without food allergies (88). Tan et al. also reported a decrease in the abundance of *A. muciniphila* in patients with psoriasis (89). In a study comparing the intestinal dysbiosis between CaOx dihydrate (COD) and monohydrate (COM) lithiasis, a large decrease in the mean values of the mucin-degrading *A. muciniphila* was observed, which is significantly more intense in COD than in COM lithiasis (90). Vakili et al. highlighted an increase in levels of *A. muciniphila* in patients with clostridium difficile infection (CDI) (86).

A decrease in *A. muciniphila* levels is also associated with many psychological disorders. For example, a study in children with autism showed a decrease in levels of *A. muciniphila* and *Bifidobacteria* species when compared with unaffected children (91). Another study showed that the abundance of *A.muciniphila* is reduced in ulcerative colitis patients suffering from depression, revealing a potential connection between psychological disorders and gut bacteria *via* the gut-brain axis (111) (**Table 2**). Finally, the protein Amuc_1100 was shown to have an antidepressant role in a chronic unpredictable mild stress (CUMS) mice model by down-regulating the brain-derived neurotrophic factor (BDNF) and inflammation in the hippocampus (112).

## *A. muciniphila*: A new probiotic?

### The development of A. muciniphila for clinical use

**Figure 2:**
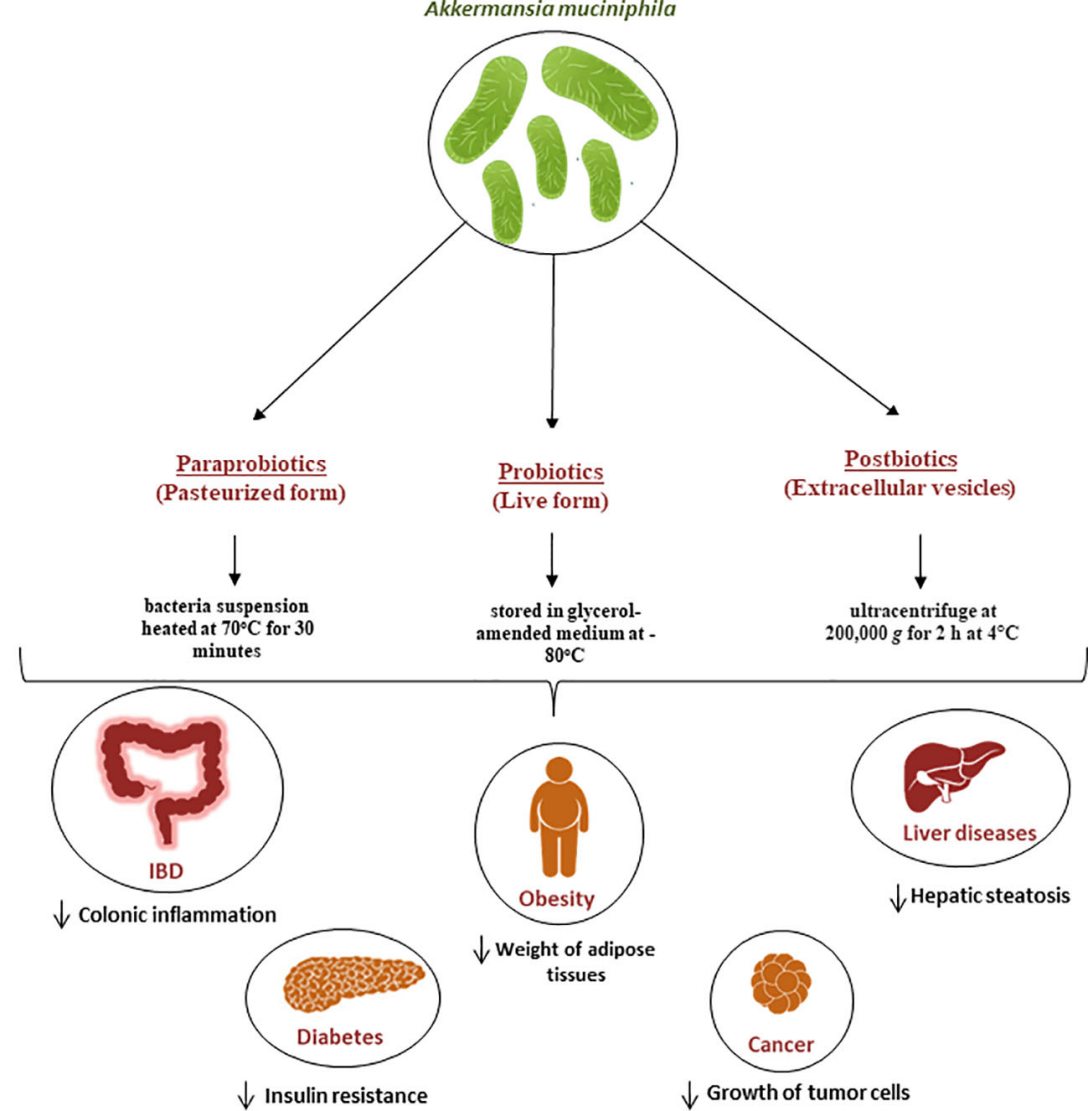
Role of *A. muciniphila* as a next generation probiotic for various metabolic diseases. Since its isolation in 2004, It has been demonstrated that *A. muciniphila* is crucial for the immune and metabolic systems regulation. It is now regarded as a “next-generation probiotic” for cancer and metabolic diseases including diabetes, liver diseases and obesity. Additionally, the pasteurized form known as parabiotic and the extracellular vesicles known as postbiotics are currently being used for the same diseases and have proven to be efficient in the treatment of these diseases.

The consumption of certain beneficial microbes, known as probiotics, has been known to affect the gut microbiota. This is because the consumption of these organisms can trigger a variety of health benefits for the host (113). It has been noted that most of the probiotics sold on the market are microorganisms from the *Bifidobacterium* and *Lactobacillus* genera (114). They are safe to use and approved by the United States Food and Drug Administration (FDA) (115). Recently, however, new microbes identified by next generation sequencing methods are emerging and are also associated with health promotion. The safety of these microbes, called next generation probiotics (NGPs), as well as their formulation and administration are currently being processed (115). *A. muciniphila* has emerged as a potential NGPs due to its various benefits on health (116). For this purpose, an efficient and scalable workflow has been developed for the cultivation and preservation of *A. muciniphila* cells. This study resulted in viable *Akkermansia* colonies with high yields and stability, with a survival up to 97.9 ± 4.5% for one year if stored in glycerol-amended medium at -80°C (117) (**Figure 2**).

In recent years, there has been a lot of focus on the use of nonviable bacterial supplements (pasteurized forms) known as paraprobiotics (118) (**Figure 2**) as an alternative to live bacteria to lower the risk of infection. for example Druart et al. demonstrated that pasteurized *A. muciniphila* is safe to use as a food ingredient based on rat models (119). The safety of *A. muciniphila* products has also been recently reported in humans (12, 107). The pasteurized form is achieved when the bacteria suspension was heated at 70°C for 30 minutes, as described by Plovier et al. (12) By comparing the effects of live and pasteurized *A. muciniphila* on normal diet-fed mice, Ashrafian et al. showed that both forms of *A. muciniphila* could modulate lipid and immune homeostasis and improved health by modulating gut microbiota, while all these effects were dominantly observed in the pasteurized form (120). Another study conducted by Grajeda-Iglesias et al. demonstrated that pasteurized *A. muciniphila* was more efficient than the live version in elevating the intestinal concentrations of polyamines, short-chain fatty acids, 2-hydroxybutyrate, as well multiple bile acids. All these metabolites have been described to be associated with human health (121). Recent studies also started focusing on postbiotics, which refers to using inactivated cell components to promote health (122). In the case of *A.muciniphila*, many studies started focusing on the potential use of its extracellular vesicles (EVs) as postbiotics (**Figure 2**). For example, a study by Ghaderi et al. showed that live and pasteurized forms of *A.muciniphila* and its EVs can affect the expression of the endocannabinoid system and peroxisome proliferator-activated receptors (PPARs) genes involved in metabolic pathways, suggesting the potential possibility to use them as probiotic, parabiotic and postbiotic respectively in order to prevent metabolic diseases (123). Furthermore, *in vitro* study showed that treatment with *A. muciniphila* or its EVs could influence the expression of genes involved in the serotonin system and thus can be used as a serotonin modulation therapy (124).

### Mice models

Many studies have focused on the causal link between *A. muciniphila* and improvements in metabolism (**Figure 2**). It has been shown that daily oral supplementation with live *A. muciniphila* at the onset of obesity, diabetes and gut barrier dysfunction in mice at the dose of 2.10^8^ bacterial cells per day improves glucose tolerance, reduces adiposity and inflammation, therefore partly protecting against diet-induced obesity in mice (108, 125). In addition, animals receiving live *A. muciniphila* no longer exhibited insulin resistance, nor infiltration of inflammatory cells (CD11c) in the adipose tissue, which is a key characteristic of obesity and associated low-grade inflammation (108). In addition, it was noted that live *A. muciniphila* prevented the development of metabolic endotoxemia as an effect associated with the restoration of a normal mucus layer thickness (108). It is worth mentioning that all these findings have subsequently been confirmed by different groups and extended to other specific disorders such as atherosclerosis, hepatic inflammation and hypercholesterolemia (20, 126, 127). Furthermore, the administration of pasteurized *A. muciniphila* was correlated with an increase in energy expenditure in diet-induced obese mice, possibly explaining the mechanism by which administration of *A. muciniphila* can reduce body weight and fat mass gain (128).

The role and administration of *A. muciniphila* have been notably investigated in cancer. For instance, a study using prostate cancer mice model showed that the extracellular vesicles of *A. muciniphila* can be used as an immunotherapeutic agent for prostate cancer treatment, demonstrated by the decrease in tumors and the upregulation of immune cells such as tumor-killing M1 macrophages after injection of these vesicles in cancer bearing mice (129). Furthermore, the effect of the mucin degrading enzyme of *A. muciniphila* Amuc_1434* (130) was investigated in the inhibition of the proliferation of CRC tissues. The study has showed that the mucin degrading enzyme Amuc_1434* was able to inhibit the proliferation of CRC cells lines by mediating apoptosis *via* the TRAIL pathway (131). However, Wang et al. suggested that treatment of CRC-mice with *A. muciniphila* increases the early level of inflammation and proliferation of the intestinal cells and therefore promotes the formation of tumors (132).

In liver diseases, recent studies have investigated the potential anti-fibrotic effects of heat-killed *A. muciniphila* Muc^T^ on the activation of hepatic stellate cell (HSC), where they demonstrated that heat-killed *A. muciniphila* Muc^T^ was safe and capable of improving LPS-induced HSC activation by modulating fibrosis markers (133). Moreover, oral supplementation in alcoholic steatohepatitis mice model induced a reduction in hepatic injury and steatosis, while enhancing mucus thickness and tight-junction expression (20). In an induced liver fibrosis mice model, it was revealed that treatment with live or pasteurized *A. muciniphila* or with its extracellular vesicles (EVs) can improve gut permeability, attenuating the expression of inflammatory biomarkers and subsequently preventing liver injury in treated mice (134). Another study showed that oral administration of *A. muciniphila* or its EVs could improve the anti-inflammatory responses eventually leading to a prevention from liver injury in mice (135). A recent study conducted by Rao et al. in mice explored the therapeutic effect of *A. muciniphila* in metabolic dysfunction-associated fatty liver disease (MAFLD). The study results indicated that *A. muciniphila* exhibited anti-MAFLD activity correlated with lipid oxidation and an improvement in gut-liver interactions by regulating the metabolism of L-aspartate (136).

The importance of *A. muciniphila* in maintaining good health and the negative correlation between its presence and obesity (81, 107) have initiated many studies focusing on using the mucin-degrading bacteria as a treatment. For example, studies performed on high-fat diet-fed mice models treated with *A. muciniphila* showed that this treatment prevents body weight gain, calorie intake and reduces the weight of adipose tissues, thus improving the induced metabolic disorders. In addition, it had many other beneficial effects such as improving glucose homeostasis and insulin sensitivity, inhibition of intestinal inflammation and restoration of damaged gut integrity (108, 137, 138). Administration of the pasteurized form had similar effects. A study by Ashrafian et al. showed that the pasteurized *A. muciniphila* and its EVs totally reduced the High-fat diet (HFD) induced intestinal inflammation and preserved intestinal permeability (139). Pasteurized *A. muciniphila* was shown to attenuate inflammatory response and improve intestinal barrier integrity. This is probably due to stimulating AMP-activated protein kinase (AMPK) and inhibiting Nuclear Factor-Kappa B (NF-κB) activation through the stimulation of TLR2 on intestinal epithelial cells (140). Aiming to understand the mechanisms of *A. muciniphila* involved in modulating the host metabolism, Yoon et al. identified a protein named P9 which induces glucagon-like peptide-1 (GLP-1) secretion and brown adipose tissue thermogenesis (141). Ashrafian et al. demonstrated that *A. muciniphila* or its EVs significantly reduced the body and fat weight of HFD mice and improved intestinal barrier integrity and energy balance (142).

Another study has been conducted to prove that deficiency in *A. muciniphila* is correlated with a high incidence of diabetes in a NOD mouse model. This study showed that the oral transfer of *A.muciniphila* could delay the onset of diabetes through promoting mucus production, increasing the expression of antimicrobial peptide Reg3y, lowering serum endotoxin levels and the expression of islet toll-like receptor (143). Chellakot et al. found that the administration of *A. muciniphila* EVs improved intestinal tight junction function, glucose tolerance in high-fat diet-induced diabetic mice and reduced weight gain, indicating a potential role for EVs in diabetes and thus indicating its use as a therapy (144).

The therapeutic role of *A. muciniphila* has also been studied in inflammatory bowel diseases. It has been found that treatment of ulcerative colitis dextran sulfate sodium (DSS)-induced mice with metformin alleviates the phenotype associated with an increase in the expression of mucin2 and in the abundance of *A. muciniphila* compared to the control group. Moreover, the administration of *A. muciniphila* decreases disruption of the mucus barrier and colonic inflammation (145). Similarly, another study conducted on a colitis DSS-induced mice model showed that the oral application of EVS protects against colitis phenotypes, such as body weight loss and inflammatory cell infiltration of the colon wall (146). Similar effects were also observed after treatment of the mice model with Amuc_2109, a β-acetylaminohexosidase secreted by *A. muciniphila*. Treatment with Amuc-2109 also had anti-inflammatory effects by inhibiting the expression of inflammatory cytokines (147).

It was found that the outer membrane protein Amuc_1100 of *A. muciniphila* promotes the biosynthesis of 5-HT, which is a neurotransmitter and a key signal molecule regulating the gastrointestinal tract functions and other organs (112). Wang et al. found that *A. muciniphila* or Amuc_1100 improved gastrointestinal motility function and restored gut microbiota abundance and species diversity in antibiotic-treated mice. This finding represented an important approach through which *A. muciniphila* interacts with the host and further influences 5-HT-related physiological functions (148).

The anti-inflammatory and immunoregulatory roles of *A. muciniphila* has been assessed in other diseases in mice models. It was shown that the administration of pasteurized *A. muciniphila* in a mouse model of H7N9 influenza viral infection reduced mortality, given its anti-inflammatory and immunoregulatory roles (149). Likewise, the administration of *A. muciniphila* resulted in a decrease in inflammatory cell infiltration and bone destruction in a mouse model of calvarial infection (49). Treatment with *A. muciniphila* also resulted in decreased alveolar bone and systemic inflammation loss in an experimental *Porphyromonas gingivalis* induced periodontitis model (49, 150). These findings highlight the protective effects of *A. muciniphila* and its use as a potential therapeutic agent to various diseases. However, Lawenius et al. showed that treatment with pasteurized *A. muciniphila* in mice reduces the accumulation of fat mass but does not protect against bone loss in a model of ovariectomized mice (151).

Moreover, another study performed in mice has reported that the presence of *A. muciniphila* and its EVs in the gut promote serotonin concentration, and also has an impact on serotonin signaling/metabolism through the gut-brain axis. These results suggest that *A. muciniphila* and its EVs can be considered as a new therapy for serotonin-related disorders (152). Ding et al. demonstrated that treatment of mice with depression induced by chronic restraint stress with *A. muciniphila* can reduce the depressive-like behavior of the mice, which was correlated with the increase in β-alanyl-3-methyl-l-histidine and edaravone (153).

### Human trials

Few studies on the use of *A. muciniphila* as a probiotic in humans have been conducted. The study by Plovier et al. was the first to demonstrate that the administration of live or pasteurized *A. muciniphila* is safe in humans in a cohort of 20 subjects with excess body weight. An exploratory study conducted by Depommier et al. on 32 overweight and obese insulin-resistant human volunteers also demonstrated that daily oral supplementation with either live or pasteurized *A. muciniphila* bacteria was safe and well-tolerated up for three months. Furthermore, they showed that pasteurized *A. muciniphila* improves insulin sensitivity and reduces insulinemia and plasma total cholesterol, while slightly decreasing body weight and fat mass compared to a placebo group (23). Moreover, the same team suggested that peroxisome proliferator-activated receptor alpha activation by mono-palmitoyl-glycerol might underlie some of the beneficial metabolic effects induced by A*. muciniphila* in human metabolic syndrome (24). Metabolome analysis illustrates that administration of *A. muciniphila* in prediabetic individuals leads to a decrease in some amino acids (tyrosine and phenylalanine), potentially explaining its hepato-protective role (154). Two clinical studies are ongoing to prove the efficacy of pasteurized *A. muciniphila* in improving insulin sensitivity, and to assess the weight-loss and glucose-lowering effects of *A. muciniphila* WST01 strain in overweight or obese patients with type 2 diabetes (**Table 3**).

**Table 3:** Human studies or clinical trials on the use of *A. muciniphila* as a probiotic or enhancing its abundance through prebiotic administration.

		Prebiotic/Probiotic	Intervention	Cohort	Clinical case	Outcomes	Author/References
**Validly published studies**	**Prebiotics**	Xylo-oligosaccharides (XOS)	1.4 g XOS, 2.8 g XOS or placebo taken daily	32 healthy subjects	x	↑ in *Akkermansia* sp. in those supplemented with the higher dose	(155)
		Resistant starch (RS)	Participants consumed a high (HC) or low carbohydrate (LC) diet followed by a baseline diet. *HC subjects consumed either a high RS (HRS – 66 g/d) or low RS (LRS – 4 g/d). *LC Subjects consumed either 48 g for HRS or 3 g for LRS.	39 subjects with reduced insulin sensitivity	x	↑ in the ratio of Firmicutes to Bacteroidetes. ↑ levels of *A. muciniphila*	(156)
	**Probiotics/Postbiotics**	*A. muciniphila*	Oral administration of either live or pasteurized *A. muciniphila* or the membrane protein Amuc_1100* (1.5 × 10^8^ CFU)	20 subjects with excess body weight	Obesity and type 2 diabetes	Administration of live or pasteurized *A. muciniphila* is safe in humans.	(12)
		*A. muciniphila*	daily oral supplementation of 10^10^ A*. muciniphila* bacteria either live or pasteurized (3 months) (10^10^ bacteria)	32 overweight/obese insulin-resistant volunteers	Obesity	1- *A. muciniphila* is safe and well tolerated. 2- Pasteurized *A. muciniphila* improved insulin sensitivity, reduced insulinemia and total plasma cholesterol. 3- Pasteurized *A. muciniphila* slightly decreased body weight and fat mass 4- *A. muciniphila* reduced the levels of markers for liver dysfunction and inflammation.	(23) (24)
		*Lactobacillus plantarum*, *Streptococus thermophiles*, *Lactobacillus acidophilus*, *Lactobacillus rhamnosus*, *Bifidobacterium lactis*, *Bifidobacterium longum*, and *Bifidobacterium breve*.	Oral supplementation (6 weeks)	13 individuals	Obesity	Increase in the abundance of *A. muciniphila* after the intervention	(157)
**Clinical trials in progress**	**Prebiotics**	oat β-glucans	5 gr of oat ß-glucan (12 weeks)	40 participants with type 2 diabetes mellitus	Type 2 diabetes	Follow up on *A. muciniphila* levels in fecal microbiota using (qPCR)	NCT04299763
		dietary fiber formulation	supplementation with 15g/day fiber powder (1 month)	20 healthy participants	x	Explore the change in *A. muciniphila* gut abundance.	NCT03785860
		Acetate	Supplementation with Acetate (Apple Cider Vinegar) (5 months).	10 patients on stable dose of antipsychotic medication for treatment of depression or anxiety.	Depression/anxiety	Encourage the growth of *A. muciniphila*	NCT05022524
		Camu Camu Capsules (CC)	2 capsules of Camu Camu daily in addition to antiretroviral therapy (12 weeks)	22 participant with HIV	HIV	1- Monitor *A. muciniphila* levels in stools. 2- Monitor gut damage and inflammation.	NCT04058392
			CC supplementation of 500 mg (3 months)	45 participants with Non-Small Cell Lung Cancer and melanoma receiving Immune Checkpoint Inhibitors	Non-Small Cell Lung Cancer and melanoma	1- Assess the safety and tolerability of CC prebiotic. 2- Discover if CC has the potential to enrich *A. muciniphila* and improve Immune Checkpoint Inhibitors efficacy.	NCT05303493
	**Probiotics**	*Lactobacillus rhamnosus* Probio-M9	Daily oral dose (6 months)	46 patients receiving immunotherapy for liver cancer	liver cancer	Increase the abundance of *A. muciniphila.*to improve effect of immunotherapy	NCT05032014
		*Lactobacillus Bifidobacterium* V9		46 Non-small Cell Lung Cancer Patients receiving immunotherapy	Cell Lung Cancer		NCT05094167
		fecal microbiota capsules	x	20 participants with Advanced Lung Cancer Treated With Immunotherapy	Advanced lung cancer	1- Selection of donor of fecal microbiota based on their fecal abundance in *F. prausnitzii, B. longum*, *A. muciniphila* and *Fusobacterium* spp. 2- Manipulating the microbial populations to enhance the efficacy of immunotherapy.	NCT04924374
		DS-01 (microbial consortia consisting of 24 strains across 12 species)	2 capsules daily (12 weeks)	100 men or women with IBS with constipation	Irritable bowel syndrome	Evaluate changes of *A. muciniphila* and other species	NCT04598295
		*A. muciniphila*	orally given *A. muciniphila* WST01 strain powder with maximum live bacteria of 5*10^10 CFU/g (12 weeks)	60 overweight/obese and drug naïve type 2 diabetes patients	Obesity/Type 2 diabetes	Evaluate the effects of *A. muciniphila* WST01 strain in overweight or obese patients with T2D.	NCT04797442
		*A. muciniphila*	Daily oral dose of pasteurized *A. muciniphila* (120 days)	98 hyperglycaemic healthy adults	Dysglycaemia	demonstrate the efficacy of pasteurized *A. muciniphila* (pAKK) in improving insulin sensitivity	NCT05114018

Table resuming the different validly published studies and the clinical trials in progress that use *A. muciniphila* as a probiotic or prebiotics to enhance its abundance, the cohort, the intervention, the clinical case, the results and the references. ↑: Increase.

### Enhancing the abundance of *A. muciniphila* with prebiotics/other probiotics

One method of favorably modulating the gut microbiota is to administer growth-promoting substrates that can be used preferentially by health-promoting bacteria to promote their growth and the production of associated desirable metabolites. The rationale of selectively enhancing beneficial microbes in the gut led to the concept of prebiotics, initially described in 1995 by Roberfroid and Gibson (158).

While some technological and regulatory hurdles may limit the use of certain strains of probiotics, it should be possible to use prebiotics and other dietary components to selectively enhance their growth in situ. The prebiotic paradigm has shifted in recent years, following the discovery of newly identified putatively beneficial gut microbiota members to target for enrichment. Through the development of new cultivation techniques and high-throughput sequencing, these studies have been able to explore the various impacts of specific fibers and products which represent untapped source of food bioactive on gut microbiota (159). For example, Anhe et al. showed that cranberry extract, rich in polyphenols, has been shown to improve diet-induced obesity and several features of metabolic syndrome (MetS) in mice, while increasing the abundance of *A. muciniphila* (160). Moreover, studies demonstrated that supplementation with grape polyphenols can promote increased intestinal abundance of *A. muciniphila* in mice fed either high-fat or low-fat diet, thus resulting in lower intestinal and systemic inflammation (161, 162). The administration of polymeric procyanidins in mice fed a high-fat/high-sucrose diet increases the proportion of *A. muciniphila* by eight times, producing beneficial effects on metabolic homeostasis (163). Another interesting fruit extract rich in polyphenols is camu-camu extract. This prebiotic can also improve the homeostasis of glucose and lipids while also increasing the abundance of *A. muciniphila* after five weeks of supplementation in HFD fed mice (164). Dietary supplementation with polysaccharides such as fucoidan decreased body weight in HFD-fed mice and also improved glucose intolerance and insulin resistance. Both fucoidans separately improved intestinal dysbiosis caused by a HFD and significantly increased the abundance of *A. muciniphila* (165). Inulin-type fructan prebiotics were found to significantly enhance the presence of *A. muciniphila*, linked to a decrease in obesity and fat mass and an improvement in insulin resistance in genetic obese and diet-induced leptin-resistant mice (166). An increase in the cecal content of *A. muciniphila* was detected by targeted qPCR following four weeks’ supplementation with berberine in genetically obese mice, associated with an improvement in gut barrier function and hepatic inflammatory and oxidative stress (167). (167) Jiang et al. showed that total flavone (TFA) extracted from the flowers Abelmoschus manihot (TFA) can also enhance *A. muciniphila* in DSS-induced experimental colitis (168). Finally, dry extract of rhubarb root has also been shown to cause an increase in levels of *A. muciniphila* associated with the increased expression of *Reg3γ* in the colon, an anti-microbial peptide with an important role in the host defense system, thus protecting against metabolic disorders (169).

Clinical trials and human studies are essential when assessing the benefits of newly identified prebiotics. Of the many potential prebiotics which have been studied, only a few substrates, including Xylo-oligosaccharides (XOS) and resistant starch (RS) have been validated through human studies. Finegold et al. demonstrated that xylo-oligosaccharides promoted intestinal health by modulating the microbial community: an increase in the levels of *Faecalibacterium* sp. and *Akkermansia* sp. as well as *Bifidobacteria* was detected (155). Moreover, a randomized dietary study by Maier et al. proved that resistant starch increased the levels of *A. muciniphila* in participants who followed a high resistant starch diet (156). Other ongoing clinical studies involve the use of various prebiotics in different diseases such as T2D, cancer and other diseases in order to uncover their potential in enriching the abundance of *A. muciniphila*.

Other probiotic treatments may also increase the levels of *A. muciniphila*. For example, a fasting programme combined with laxative treatment for one week followed by a six-week probiotic intervention with a probiotic containing several different bacterial strains showed an increase in the abundance of *Akkermansia* (157). Four other clinical studies are in progress about the use of different bacteria as probiotics and their effect on modulation of the intestinal flora in cancer or IBS and, most importantly, on increasing the abundance of *A. muciniphila* (**Table 3**).

Other than natural components, *A. muciniphila* has been used by Payahoo et al. as a marker to assess the efficiency of a pharmaceutical agent, Oleoylethanolamide, for treatment of obese people. This study showed that abundance of *A. muciniphila* bacterium increases significantly in oleoylethanolamide group compared to the placebo group and modifies the energy balance (170).

## Conclusion and perspectives

A new area of research is emerging with the study of interbacterial communication, particularly between probiotic bacteria in transit and intestinal bacteria. *A. muciniphila* has been proven to have many beneficial effects in immune and metabolic regulation which can result in stimulating host health and preventing of pathogens. Nowadays, it is considered as a next generation probiotic to treat metabolic disorders such as obesity, diabetes, inflammatory diseases, as well as cancer (**Figure 2**). It has been reported that *A. muciniphila* in its two forms (live and pasteurized) is safe for use in human trials and two known companies have already started producing *A. muciniphila* probiotics (A-Mansia Biotech and Pendulum). However, there is no significant evidence on the link between this bacteria and malnutrition, reason why more studies should focus on this topic. Finally, more studies and mainly human clinical trials should be carried out in order to assess mechanisms of action and long-term effects of *A. muciniphila* before using for therapeutic applications.

## Author contributions

RI, RW: Writing-original draft preparation. GD: Writing, reviewing and editing. J-CL, DR: Reviewing, supervision. All authors have read and agreed to the published version of the manuscript. All authors contributed to the article and approved the submitted version.

## Funding

This study was supported by the Institut Hospitalo-Universitaire (IHU) Méditerranée Infection, the National Research Agency under the “Investissements d’avenir” programme, reference ANR-10-IAHU-03, the Région Provence Alpes Côte d’Azur and European ERDF PRIMI funding.

## Conflict of interest

The authors declare that the research was conducted in the absence of any commercial or financial relationships that could be construed as a potential conflict of interest.

## Publisher’s note

All claims expressed in this article are solely those of the authors and do not necessarily represent those of their affiliated organizations, or those of the publisher, the editors and the reviewers. Any product that may be evaluated in this article, or claim that may be made by its manufacturer, is not guaranteed or endorsed by the publisher.

## Supplementary material

The Supplementary Material for this article can be found online at: https://www.frontiersin.org/articles/10.3389/fgstr.2022.1024393/full#supplementary-material



## References

[1] Canakis A Haroon M Weber HC. Irritable bowel syndrome and gut microbiota. Curr Opin Endocrinol Diabetes Obes (2020) 27:28–35. doi: 10.1097/MED.0000000000000523 31789724

[2] Gurung M Li Z You H Rodrigues R Jump DB Morgun A. Role of gut microbiota in type 2 diabetes pathophysiology. EBioMedicine (2020) 51:102590. doi: 10.1016/j.ebiom.2019.11.051 31901868 PMC6948163

[3] Zitvogel L Galluzzi L Viaud S Vétizou M Daillère R Merad M. Cancer and the gut microbiota: An unexpected link. Sci Transl Med (2015) 7:102590. doi: 10.1126/scitranslmed.3010473 PMC4690201 25609166

[4] Lam Y Maguire S Palacios T Caterson I. Are the gut bacteria telling us to eat or not to eat? Reviewing the role of gut microbiota in the etiology, disease progression and treatment of eating disorders. Nutrients (2017) 9:602. doi: 10.3390/nu9060602 28613252 PMC5490581

[5] Liang S Wu X Jin F. Gut-brain psychology: Rethinking psychology from the microbiota–Gut–Brain axis. Front Integr Neurosci (2018) 12:33. doi: 10.3389/fnint.2018.00033 30271330 PMC6142822

[6] Rinninella E Raoul P Cintoni M Franceschi F Miggiano G Gasbarrini A. What is the healthy gut microbiota composition? A changing ecosystem across age, environment, diet, and diseases. Microorganisms (2019) 7:14. doi: 10.3390/microorganisms7010014 30634578 PMC6351938

[7] Derrien M Vaughan EE Plugge CM de Vos WM. Akkermansia muciniphila gen. nov., sp. nov., a human intestinal mucin-degrading bacterium. Int J Syst Evol Microbiol (2004) 54:1469–76. doi: 10.1099/ijs.0.02873-0 15388697

[8] Collado MC Derrien M Isolauri E de Vos WM Salminen S. Intestinal integrity and *Akkermansia muciniphila*, a mucin-degrading member of the intestinal microbiota present in infants, adults, and the elderly. Appl Environ Microbiol (2007) 73:7767–70. doi: 10.1128/AEM.01477-07 PMC2168041 17933936

[9] Derrien M Collado MC Ben-Amor K Salminen S de Vos WM. The mucin degrader *Akkermansia muciniphila* is an abundant resident of the human intestinal tract. Appl Environ Microbiol (2008) 74:1646–8. doi: 10.1128/AEM.01226-07 PMC2258631 18083887

[10] Ottman N Geerlings SY Aalvink S de Vos WM Belzer C. Action and function of akkermansia muciniphila in microbiome ecology, health and disease. Best Pract Res Clin Gastroenterol (2017) 31:637–42. doi: 10.1016/j.bpg.2017.10.001 29566906

[11] Reunanen J Kainulainen V Huuskonen L Ottman N Belzer C Huhtinen H. Akkermansia muciniphila adheres to enterocytes and strengthens the integrity of the epithelial cell layer. Appl Environ Microbiol (2015) 81:3655–62. doi: 10.1128/AEM.04050-14 PMC4421065 25795669

[12] Plovier H Everard A Druart C Depommier C Van Hul M Geurts L. A purified membrane protein from akkermansia muciniphila or the pasteurized bacterium improves metabolism in obese and diabetic mice. Nat Med (2017) 23:107–13. doi: 10.1038/nm.4236 27892954

[13] Ottman N Reunanen J Meijerink M Pietilä TE Kainulainen V Klievink J. Pili-like proteins of akkermansia muciniphila modulate host immune responses and gut barrier function. PloS One (2017) 12:e0173004. doi: 10.1371/journal.pone.0173004 28249045 PMC5332112

[14] Zhou Q Zhang Y Wang X Yang R Zhu X Zhang Y. Gut bacteria akkermansia is associated with reduced risk of obesity: Evidence from the American gut project. Nutr Metab (Lond) (2020) 17:90. doi: 10.1186/s12986-020-00516-1 33110437 PMC7583218

[15] Vernocchi P Gili T Conte F Del Chierico F Conta G Miccheli A. Network analysis of gut microbiome and metabolome to discover microbiota-linked biomarkers in patients affected by non-small cell lung cancer. IJMS (2020) 21:8730. doi: 10.3390/ijms21228730 33227982 PMC7699235

[16] Osman MA Neoh H Ab Mutalib N-S Chin S-F Mazlan L Raja Ali RA. Parvimonas micra, peptostreptococcus stomatis, fusobacterium nucleatum and akkermansia muciniphila as a four-bacteria biomarker panel of colorectal cancer. Sci Rep (2021) 11:2925. doi: 10.1038/s41598-021-82465-0 33536501 PMC7859180

[17] Li N Bai C Zhao L Ge Y Li X. Characterization of the fecal microbiota in gastrointestinal cancer patients and healthy people. Clin Transl Oncol (2022) 11:2925. doi: 10.1007/s12094-021-02754-y 35167015

[18] Zhang J Ni Y Qian L Fang Q Zheng T Zhang M. Decreased abundance of *Akkermansia muciniphila* leads to the impairment of insulin secretion and glucose homeostasis in lean type 2 diabetes. Adv Sci (2021) 8:2100536. doi: 10.1002/advs.202100536 PMC8373164 34085773

[19] Earley H Lennon G Balfe Á Coffey JC Winter DC O’Connell PR. The abundance of akkermansia muciniphila and its relationship with sulphated colonic mucins in health and ulcerative colitis. Sci Rep (2019) 9:15683. doi: 10.1038/s41598-019-51878-3 31666581 PMC6821857

[20] Grander C Adolph TE Wieser V Lowe P Wrzosek L Gyongyosi B. Recovery of ethanol-induced *Akkermansia muciniphila* depletion ameliorates alcoholic liver disease. Gut (2018) 67:891–901. doi: 10.1136/gutjnl-2016-313432 28550049

[21] Cani PD de Vos WM. Next-generation beneficial microbes: The case of akkermansia muciniphila. Front Microbiol (2017) 8:1765. doi: 10.3389/fmicb.2017.01765 29018410 PMC5614963

[22] Zhai Q Feng S Arjan N Chen W. A next generation probiotic, *Akkermansia muciniphila* . Crit Rev Food Sci Nutr (2019) 59:3227–36. doi: 10.1080/10408398.2018.1517725 30373382

[23] Depommier C Everard A Druart C Plovier H Van Hul M Vieira-Silva S. Supplementation with akkermansia muciniphila in overweight and obese human volunteers: a proof-of-concept exploratory study. Nat Med (2019) 25:1096–103. doi: 10.1038/s41591-019-0495-2 PMC6699990 31263284

[24] Depommier C Vitale RM Iannotti FA Silvestri C Flamand N Druart C. Beneficial effects of akkermansia muciniphila are not associated with major changes in the circulating endocannabinoidome but linked to higher mono-Palmitoyl-Glycerol levels as new PPARα agonists. Cells (2021) 10:185. doi: 10.3390/cells10010185 33477821 PMC7832901

[25] Ouwerkerk JP Aalvink S Belzer C de Vos WM. Akkermansia glycaniphila sp. nov., an anaerobic mucin-degrading bacterium isolated from reticulated python faeces. Int J Syst Evol Microbiol (2016) 66:4614–20. doi: 10.1099/ijsem.0.001399 27499019

[26] Lv Q-B Li S-H Zhang Y Wang Y-C Peng Y-Z Zhang X-X. A thousand metagenome-assembled genomes of *Akkermansia* reveal new phylogroups and geographical and functional variations in human gut. [preprint]. Genomics (2020) 66:4614–20. doi: 10.1101/2020.09.10.292292 PMC9378777 35982777

[27] van Passel MWJ Kant R Zoetendal EG Plugge CM Derrien M Malfatti SA. The genome of akkermansia muciniphila, a dedicated intestinal mucin degrader, and its use in exploring intestinal metagenomes. PloS One (2011) 6:e16876. doi: 10.1371/journal.pone.0016876 21390229 PMC3048395

[28] Xing J Li X Sun Y Zhao J Miao S Xiong Q. Comparative genomic and functional analysis of akkermansia muciniphila and closely related species. Genes Genom (2019) 41:1253–64. doi: 10.1007/s13258-019-00855-1 PMC6828834 31399846

[29] Karcher N Nigro E Punčochář M Blanco-Míguez A Ciciani M Manghi P. Genomic diversity and ecology of human-associated akkermansia species in the gut microbiome revealed by extensive metagenomic assembly. Genome Biol (2021) 22:209. doi: 10.1186/s13059-021-02427-7 34261503 PMC8278651

[30] Guo X Li S Zhang J Wu F Li X Wu D. Genome sequencing of 39 akkermansia muciniphila isolates reveals its population structure, genomic and functional diverisity, and global distribution in mammalian gut microbiotas. BMC Genomics (2017) 18:800. doi: 10.1186/s12864-017-4195-3 29047329 PMC5648452

[31] Kirmiz N Galindo K Cross KL Luna E Rhoades N Podar M. Comparative genomics guides elucidation of vitamin b _12_ biosynthesis in novel human-associated *Akkermansia* strains. Appl Environ Microbiol (2020) 86:800. doi: 10.1128/AEM.02117-19 PMC6974653 31757822

[32] Becken B Davey L Middleton DR Mueller KD Sharma A Holmes ZC. Genotypic and phenotypic diversity among human isolates of akkermansia muciniphila. mBio (2021) 12:e02117–19. doi: 10.1128/mBio.00478-21 PMC8262928 34006653

[33] Ottman N Davids M Suarez-Diez M Boeren S Schaap PJ Martins dos Santos VAP. Genome-scale model and omics analysis of metabolic capacities of *Akkermansia muciniphila* reveal a preferential mucin-degrading lifestyle. Appl Environ Microbiol (2017) 83:e00478–21. doi: 10.1128/AEM.01014-17 PMC5583483 28687644

[34] Chia LW Hornung BVH Aalvink S Schaap PJ de Vos WM Knol J. Deciphering the trophic interaction between akkermansia muciniphila and the butyrogenic gut commensal anaerostipes caccae using a metatranscriptomic approach. Antonie van Leeuwenhoek (2018) 111:859–73. doi: 10.1007/s10482-018-1040-x PMC5945754 29460206

[35] Kosciow K Deppenmeier U. Characterization of a phospholipid-regulated β-galactosidase from *Akkermansia muciniphila* involved in mucin degradation. MicrobiologyOpen (2019) 8:e01343–17. doi: 10.1002/mbo3.796 PMC6692548 30729732

[36] Kosciow K Deppenmeier U. Characterization of three novel β-galactosidases from akkermansia muciniphila involved in mucin degradation. Int J Biol Macromolecules (2020) 149:331–40. doi: 10.1016/j.ijbiomac.2020.01.246 31991210

[37] Dubourg G Cornu F Edouard S Battaini A Tsimaratos M Raoult D. First isolation of akkermansia muciniphila in a blood-culture sample. Clin Microbiol Infect (2017) 23:682–3. doi: 10.1016/j.cmi.2017.02.031 28274768

[38] Dubourg G Lagier J-C Armougom F Robert C Audoly G Papazian L. High-level colonisation of the human gut by verrucomicrobia following broad-spectrum antibiotic treatment. Int J Antimicrobial Agents (2013) 41:149–55. doi: 10.1016/j.ijantimicag.2012.10.012 23294932

[39] Caputo A Dubourg G Croce O Gupta S Robert C Papazian L. Whole-genome assembly of akkermansia muciniphila sequenced directly from human stool. Biol Direct (2015) 10:5. doi: 10.1186/s13062-015-0041-1 25888298 PMC4333879

[40] Mailhe M Ricaboni D Vitton V Gonzalez J-M Bachar D Dubourg G. Repertoire of the gut microbiota from stomach to colon using culturomics and next-generation sequencing. BMC Microbiol (2018) 18:157. doi: 10.1186/s12866-018-1304-7 30355340 PMC6201554

[41] Ye F Shen H Li Z Meng F Li L Yang J. Influence of the biliary system on biliary bacteria revealed by bacterial communities of the human biliary and upper digestive tracts. PloS One (2016) 11:e0150519. doi: 10.1371/journal.pone.0150519 26930491 PMC4773253

[42] Li G Yang M Zhou K Zhang L Tian L Lv S. Diversity of duodenal and rectal microbiota in biopsy tissues and luminal contents in healthy volunteers. J Microbiol Biotechnol (2015) 25:1136–45. doi: 10.4014/jmb.1412.12047 25737115

[43] Rogers MB Aveson V Firek B Yeh A Brooks B Brower-Sinning R. Disturbances of the perioperative microbiome across multiple body sites in patients undergoing pancreaticoduodenectomy. Pancreas (2017) 46:260–7. doi: 10.1097/MPA.0000000000000726 PMC5235958 27846140

[44] Rossen NG Fuentes S Boonstra K D’Haens GR Heilig HG Zoetendal EG. The mucosa-associated microbiota of PSC patients is characterized by low diversity and low abundance of uncultured clostridiales II. J Crohn’s Colitis (2015) 9:342–8. doi: 10.1093/ecco-jcc/jju023 25547975

[45] Wang M AhrnÃ S Jeppsson B Molin G. Comparison of bacterial diversity along the human intestinal tract by direct cloning and sequencing of 16S rRNA genes. FEMS Microbiol Ecol (2005) 54:219–31. doi: 10.1016/j.femsec.2005.03.012 16332321

[46] McHardy IH Goudarzi M Tong M Ruegger PM Schwager E Weger JR. Integrative analysis of the microbiome and metabolome of the human intestinal mucosal surface reveals exquisite inter-relationships. Microbiome (2013) 1:17. doi: 10.1186/2049-2618-1-17 24450808 PMC3971612

[47] Le T Chun E Lopez I Kingsley K Nguyen L. Screening for selenomonas noxia and akkermansia muciniphila from the oral cavity of pediatric patients. MRJI (2021), 28–33. doi: 10.9734/mrji/2021/v31i730331

[48] Coretti L Cuomo M Florio E Palumbo D Keller S Pero R. Subgingival dysbiosis in smoker and non-smoker patients with chronic periodontitis. Mol Med Rep (2017) 15:2007–14. doi: 10.3892/mmr.2017.6269 PMC5364964 28260061

[49] Huck O Mulhall H Rubin G Kizelnik Z Iyer R Perpich JD. *Akkermansia muciniphila* reduces *Porphyromonas gingivalis* -induced inflammation and periodontal bone destruction. J Clin Periodontol (2020) 47:202–12. doi: 10.1111/jcpe.13214 31674689

[50] Dubourg G Morand A Mekhalif F Godefroy R Corthier A Yacouba A. Deciphering the urinary microbiota repertoire by culturomics reveals mostly anaerobic bacteria from the gut. Front Microbiol (2020) 11:513305. doi: 10.3389/fmicb.2020.513305 33178140 PMC7596177

[51] Mansour B Monyók Á Makra N Gajdács M Vadnay I Ligeti B. Bladder cancer-related microbiota: examining differences in urine and tissue samples. Sci Rep (2020) 10:11042. doi: 10.1038/s41598-020-67443-2 32632181 PMC7338485

[52] Liu F Ling Z Xiao Y Yang Q Zheng L Jiang P. Characterization of the urinary microbiota of elderly women and the effects of type 2 diabetes and urinary tract infections on the microbiota. Oncotarget (2017) 8:100678–90. doi: 10.18632/oncotarget.21126 PMC5725054 29246012

[53] Martín R Heilig HGHJ Zoetendal EG Jiménez E Fernández L Smidt H. Cultivation-independent assessment of the bacterial diversity of breast milk among healthy women. Res Microbiol (2007) 158:31–7. doi: 10.1016/j.resmic.2006.11.004 17224259

[54] Collado MC Laitinen K Salminen S Isolauri E. Maternal weight and excessive weight gain during pregnancy modify the immunomodulatory potential of breast milk. Pediatr Res (2012) 72:77–85. doi: 10.1038/pr.2012.42 22453296

[55] Urbaniak C Cummins J Brackstone M Macklaim JM Gloor GB Baban CK. Microbiota of human breast tissue. Appl Environ Microbiol (2014) 80:3007–14. doi: 10.1128/AEM.00242-14 PMC4018903 24610844

[56] Aakko J Kumar H Rautava S Wise A Autran C Bode L. Human milk oligosaccharide categories define the microbiota composition in human colostrum. Benef Microbes (2017) 8:563–7. doi: 10.3920/BM2016.0185 28726512

[57] Kim SY Yi DY. Analysis of the human breast milk microbiome and bacterial extracellular vesicles in healthy mothers. Exp Mol Med (2020) 52:1288–97. doi: 10.1038/s12276-020-0470-5 PMC8080581 32747701

[58] Štšepetova J Simre K Tagoma A Uibo O Peet A Siljander H. Maternal breast milk microbiota and immune markers in relation to subsequent development of celiac disease in offspring. Sci Rep (2022) 12:6607. doi: 10.1038/s41598-022-10679-x 35459889 PMC9033794

[59] Kostopoulos I Elzinga J Ottman N Klievink JT Blijenberg B Aalvink S. Akkermansia muciniphila uses human milk oligosaccharides to thrive in the early life conditions. vitro. Sci Rep (2020) 10:14330. doi: 10.1038/s41598-020-71113-8 32868839 PMC7459334

[60] Luna E Parkar SG Kirmiz N Hartel S Hearn E Hossine M. Utilization efficiency of human milk oligosaccharides by human-associated akkermansia is strain dependent. Appl Environ Microbiol (2022) 88:e0148721. doi: 10.1128/AEM.01487-21 34669436 PMC8752153

[61] Guo X Zhang J Wu F Zhang M Yi M Peng Y. Different subtype strains of *Akkermansia muciniphila* abundantly colonize in southern China. J Appl Microbiol (2016) 120:452–9. doi: 10.1111/jam.13022 PMC4736461 26666632

[62] Adamberg K Adamberg S. Selection of fast and slow growing bacteria from fecal microbiota using continuous culture with changing dilution rate. Microbial Ecol Health Dis (2018) 29:1549922. doi: 10.1080/16512235.2018.1549922 PMC6282430 30532686

[63] Yousi F Kainan C Junnan Z Chuanxing X Lina F Bangzhou Z. Evaluation of the effects of four media on human intestinal microbiota culture. vitro. AMB Expr (2019) 9:69. doi: 10.1186/s13568-019-0790-9 PMC6533344 31123874

[64] Liu X Zhao F Liu H Xie Y Zhao D Li C. Transcriptomics and metabolomics reveal the adaption of akkermansia muciniphila to high mucin by regulating energy homeostasis. Sci Rep (2021) 11:9073. doi: 10.1038/s41598-021-88397-z 33907216 PMC8079684

[65] Li Z Hu G Zhu L Sun Z Jiang Y Gao M. Study of growth, metabolism, and morphology of akkermansia muciniphila with an *in vitro* advanced bionic intestinal reactor. BMC Microbiol (2021) 21:61. doi: 10.1186/s12866-021-02111-7 33622254 PMC7901181

[66] Ogata Y Sakamoto M Ohkuma M Hattori M Suda W. Complete genome sequence of akkermansia muciniphila JCM 30893, isolated from feces of a healthy Japanese Male. Microbiol Resour Announc (2020) 9:9073. doi: 10.1128/MRA.01543-19 PMC7019068 32054713

[67] Lagier J-C Khelaifia S Alou MT Ndongo S Dione N Hugon P. Culture of previously uncultured members of the human gut microbiota by culturomics. Nat Microbiol (2016) 1:16203. doi: 10.1038/nmicrobiol.2016.203 27819657 PMC12094094

[68] Diakite A Dubourg G Dione N Afouda P Bellali S Ngom II. Optimization and standardization of the culturomics technique for human microbiome exploration. Sci Rep (2020) 10:9674. doi: 10.1038/s41598-020-66738-8 32541790 PMC7295790

[69] Van Herreweghen F Van den Abbeele P De Mulder T De Weirdt R Geirnaert A Hernandez-Sanabria E. *In vitro* colonisation of the distal colon by *Akkermansia muciniphila* is largely mucin and pH dependent. Benef Microbes (2017) 8:81–96. doi: 10.3920/BM2016.0013 27824274

[70] Van den Abbeele P Grootaert C Marzorati M Possemiers S Verstraete W Gérard P. Microbial community development in a dynamic gut model is reproducible, colon region specific, and selective for *Bacteroidetes* and *Clostridium* cluster IX. Appl Environ Microbiol (2010) 76:5237–46. doi: 10.1128/AEM.00759-10 PMC2916472 20562281

[71] Machado D Almeida D Seabra CL Andrade JC Gomes AM Freitas AC. Uncovering akkermansia muciniphila resilience or susceptibility to different temperatures, atmospheres and gastrointestinal conditions. Anaerobe (2020) 61:102135. doi: 10.1016/j.anaerobe.2019.102135 31875576

[72] Ouwerkerk JP van der Ark KCH Davids M Claassens NJ Finestra TR de Vos WM. Adaptation of akkermansia muciniphila to the oxic-anoxic interface of the mucus layer. Appl Environ Microbiol (2016) 82:6983–93. doi: 10.1128/AEM.01641-16 PMC5103097 27663027

[73] Durand P Golinelli-Pimpaneau B Mouilleron S Badet B Badet-Denisot M-A. Highlights of glucosamine-6P synthase catalysis. Arch Biochem Biophys (2008) 474:302–17. doi: 10.1016/j.abb.2008.01.026 18279655

[74] Hagi T Geerlings SY Nijsse B Belzer C. The effect of bile acids on the growth and global gene expression profiles in akkermansia muciniphila. Appl Microbiol Biotechnol (2020) 104:10641–53. doi: 10.1007/s00253-020-10976-3 PMC7671984 33159542

[75] Kaźmierczak-Siedlecka K Skonieczna-Żydecka K Hupp T Duchnowska R Marek-Trzonkowska N Połom K. Next-generation probiotics – do they open new therapeutic strategies for cancer patients? Gut Microbes (2022) 14:2035659. doi: 10.1080/19490976.2022.2035659 35167406 PMC8855854

[76] Swidsinski A Dorffel Y Loening-Baucke V Theissig F Ruckert JC Ismail M. Acute appendicitis is characterised by local invasion with fusobacterium nucleatum/necrophorum. Gut (2011) 60:34–40. doi: 10.1136/gut.2009.191320 19926616

[77] Png CW Lindén SK Gilshenan KS Zoetendal EG McSweeney CS Sly LI. Mucolytic bacteria with increased prevalence in IBD mucosa augment *In vitro* utilization of mucin by other bacteria. Am J Gastroenterol (2010) 105:2420–8. doi: 10.1038/ajg.2010.281 20648002

[78] Leyrolle Q Cserjesi R Mulders MDGH Zamariola G Hiel S Gianfrancesco MA. Specific gut microbial, biological, and psychiatric profiling related to binge eating disorders: A cross-sectional study in obese patients. Clin Nutr (2021) 40:2035–44. doi: 10.1016/j.clnu.2020.09.025 33023763

[79] Seck EH Senghor B Merhej V Bachar D Cadoret F Robert C. Salt in stools is associated with obesity, gut halophilic microbiota and akkermansia muciniphila depletion in humans. Int J Obes (2019) 43:862–71. doi: 10.1038/s41366-018-0201-3 30206336

[80] Dao MC Belda E Prifti E Everard A Kayser BD Bouillot J-L. *Akkermansia muciniphila* abundance is lower in severe obesity, but its increased level after bariatric surgery is not associated with metabolic health improvement. Am J Physiology-Endocrinol Metab (2019) 317:E446–59. doi: 10.1152/ajpendo.00140.2019 31265324

[81] Karlsson CLJ Önnerfält J Xu J Molin G Ahrné S Thorngren-Jerneck K. The microbiota of the gut in preschool children with normal and excessive body weight. Obesity (2012) 20:2257–61. doi: 10.1038/oby.2012.110 22546742

[82] F.S.Teixeira T Grześkowiak ŁM Salminen S Laitinen K Bressan J Gouveia Peluzio M do C. Faecal levels of bifidobacterium and clostridium coccoides but not plasma lipopolysaccharide are inversely related to insulin and HOMA index in women. Clin Nutr (2013) 32:1017–22. doi: 10.1016/j.clnu.2013.02.008 23538004

[83] Allin KH Tremaroli V Caesar R Jensen BAH Damgaard MTF Bahl MI. Aberrant intestinal microbiota in individuals with prediabetes. Diabetologia (2018) 61:810–20. doi: 10.1007/s00125-018-4550-1 PMC6448993 29379988

[84] Qin J Li Y Cai Z Li S Zhu J Zhang F. A metagenome-wide association study of gut microbiota in type 2 diabetes. Nature (2012) 490:55–60. doi: 10.1038/nature11450 23023125

[85] Liu F Ling Z Xiao Y Lv L Yang Q Wang B. Dysbiosis of urinary microbiota is positively correlated with type 2 diabetes mellitus. Oncotarget (2017) 8:3798–810. doi: 10.18632/oncotarget.14028 PMC5354796 28008148

[86] Vakili B Fateh A Asadzadeh Aghdaei H Sotoodehnejadnematalahi F Siadat SD. Characterization of gut microbiota in hospitalized patients with clostridioides difficile infection. Curr Microbiol (2020) 77:1673–80. doi: 10.1007/s00284-020-01980-x 32296918

[87] Demirci M Tokman HB Uysal HK Demiryas S Karakullukcu A Saribas S. Reduced akkermansia muciniphila and faecalibacterium prausnitzii levels in the gut microbiota of children with allergic asthma. Allergol Immunopathol (2019) 47:365–71. doi: 10.1016/j.aller.2018.12.009 30765132

[88] Fieten KB Totté JEE Levin E Reyman M Meijer Y Knulst A. Fecal microbiome and food allergy in pediatric atopic dermatitis: A cross-sectional pilot study. Int Arch Allergy Immunol (2018) 175:77–84. doi: 10.1159/000484897 29393195

[89] Tan L Zhao S Zhu W Wu L Li J Shen M. The *Akkermansia muciniphila* is a gut microbiota signature in psoriasis. Exp Dermatol (2018) 27:144–9. doi: 10.1111/exd.13463 29130553

[90] Rodríguez FM Sabiote Rubio L Girón Nanne I Sánchez Martín F Emiliani E Angerri Feu O. The relationship between calcium oxalate lithiasis and chronic proinflammatory intestinal dysbiosis pattern: a prospective study. Urolithiasis (2020) 48:321–8. doi: 10.1007/s00240-020-01181-y 32107580

[91] Wang L Christophersen CT Sorich MJ Gerber JP Angley MT Conlon MA. Low relative abundances of the mucolytic bacterium akkermansia muciniphila and bifidobacterium spp. in feces of children with autism. Appl Environ Microbiol (2011) 77:6718–21. doi: 10.1128/AEM.05212-11 PMC3187122 21784919

[92] Panebianco C Adamberg K Jaagura M Copetti M Fontana A Adamberg S. Influence of gemcitabine chemotherapy on the microbiota of pancreatic cancer xenografted mice. Cancer Chemother Pharmacol (2018) 81:773–82. doi: 10.1007/s00280-018-3549-0 29473096

[93] Terrisse S Goubet A-G Ueda K Thomas AM Quiniou V Thelemaque C. Immune system and intestinal microbiota determine efficacy of androgen deprivation therapy against prostate cancer. J Immunother Cancer (2022) 10:e004191. doi: 10.1136/jitc-2021-004191 35296557 PMC8928383

[94] Miller PL Carson TL. Mechanisms and microbial influences on CTLA-4 and PD-1-based immunotherapy in the treatment of cancer: a narrative review. Gut Pathog (2020) 12:43. doi: 10.1186/s13099-020-00381-6 32944086 PMC7488430

[95] He D Li X An R Wang L Wang Y Zheng S. Response to PD-1-Based immunotherapy for non-small cell lung cancer altered by gut microbiota. Oncol Ther (2021) 9:647–57. doi: 10.1007/s40487-021-00171-3 PMC8593091 34664203

[96] Liang W Yang Y Wang H Wang H Yu X Lu Y. Gut microbiota shifts in patients with gastric cancer in perioperative period. Medicine (2019) 98:e16626. doi: 10.1097/MD.0000000000016626 31464899 PMC6736490

[97] Routy B Le Chatelier E Derosa L Duong CPM Alou MT Daillère R. Gut microbiome influences efficacy of PD-1–based immunotherapy against epithelial tumors. Science (2018) 359:91–7. doi: 10.1126/science.aan3706 29097494

[98] Derosa L Routy B Fidelle M Iebba V Alla L Pasolli E. Gut bacteria composition drives primary resistance to cancer immunotherapy in renal cell carcinoma patients. Eur Urol (2020) 78:195–206. doi: 10.1016/j.eururo.2020.04.044 32376136

[99] Hou X Zhang P Du H Chu W Sun R Qin S. Akkermansia muciniphila potentiates the antitumor efficacy of FOLFOX in colon cancer. Front Pharmacol (2021) 12:725583. doi: 10.3389/fphar.2021.725583 34603035 PMC8484791

[100] Lungulescu CV Răileanu S Afrem G Ungureanu BS Florescu DN Gheonea IA. Histochemical and immunohistochemical study of mucinous rectal carcinoma. J Med Life (2017) 10:139–43. PMC5467255 28616090

[101] Dizman N Hsu J Bergerot PG Gillece JD Folkerts M Reining L. Randomized trial assessing impact of probiotic supplementation on gut microbiome and clinical outcome from targeted therapy in metastatic renal cell carcinoma. Cancer Med (2021) 10:79–86. doi: 10.1002/cam4.3569 33135866 PMC7826461

[102] Mitsou EK Detopoulou M Kakali A Fragopoulou E Nomikos T Antonopoulou S. Mining possible associations of faecal a. muciniphila colonisation patterns with host adiposity and cardiometabolic markers in an adult population. Benef Microbes (2019) 10:741–9. doi: 10.3920/BM2019.0033 31965843

[103] Zhou J-C Zhang X-W. Akkermansia muciniphila: a promising target for the therapy of metabolic syndrome and related diseases. Chin J Natural Medicines (2019) 17:835–41. doi: 10.1016/S1875-5364(19)30101-3 31831130

[104] Nakano H Wu S Sakao K Hara T He J Garcia S. Bilberry anthocyanins ameliorate NAFLD by improving dyslipidemia and gut microbiome dysbiosis. Nutrients (2020) 12:3252. doi: 10.3390/nu12113252 33114130 PMC7690841

[105] Li S Wang N Tan H Chueng F Zhang Z Yuen M. Modulation of gut microbiota mediates berberine-induced expansion of immuno-suppressive cells to against alcoholic liver disease. Clin Trans Med (2020) 10:835–41. doi: 10.1002/ctm2.112 PMC7438809 32790968

[106] Santacruz A Collado MC García-Valdés L Segura MT Martín-Lagos JA Anjos T. Gut microbiota composition is associated with body weight, weight gain and biochemical parameters in pregnant women. Br J Nutr (2010) 104:83–92. doi: 10.1017/S0007114510000176 20205964

[107] Dao MC Everard A Aron-Wisnewsky J Sokolovska N Prifti E Verger EO. *Akkermansia muciniphila* and improved metabolic health during a dietary intervention in obesity: relationship with gut microbiome richness and ecology. Gut (2016) 65:426–36. doi: 10.1136/gutjnl-2014-308778 26100928

[108] Everard A Belzer C Geurts L Ouwerkerk JP Druart C Bindels LB. Cross-talk between akkermansia muciniphila and intestinal epithelium controls diet-induced obesity. Proc Natl Acad Sci (2013) 110:9066–71. doi: 10.1073/pnas.1219451110 PMC3670398 23671105

[109] Wang C-H Yen H-R Lu W-L Ho H-H Lin W-Y Kuo Y-W. Adjuvant probiotics of lactobacillus salivarius subsp. salicinius AP-32, l. johnsonii MH-68, and bifidobacterium animalis subsp. lactis CP-9 attenuate glycemic levels and inflammatory cytokines in patients with type 1 diabetes mellitus. Front Endocrinol (2022) 13:754401. doi: 10.3389/fendo.2022.754401 PMC8921459 35299968

[110] Cruz-Aguliar RM Wantia N Clavel T Vehreschild MJGT Buch T Bajbouj M. An open-labeled study on fecal microbiota transfer in irritable bowel syndrome patients reveals improvement in abdominal pain associated with the relative abundance of. Akkermansia Muciniphila Digest (2019) 100:127–38. doi: 10.1159/000494252 30423561

[111] Chen T Wang R Duan Z Yuan X Ding Y Feng Z. Akkermansia muciniphila protects against psychological disorder-induced gut microbiota-mediated colonic mucosal barrier damage and aggravation of colitis. Front Cell Infect Microbiol (2021) 11:723856. doi: 10.3389/fcimb.2021.723856 34722332 PMC8551916

[112] Cheng R Xu W Wang J Tang Z Zhang M. The outer membrane protein Amuc_1100 of akkermansia muciniphila alleviates the depression-like behavior of depressed mice induced by chronic stress. Biochem Biophys Res Commun (2021) 566:170–6. doi: 10.1016/j.bbrc.2021.06.018 34129964

[113] Hill C Guarner F Reid G Gibson GR Merenstein DJ Pot B. The international scientific association for probiotics and prebiotics consensus statement on the scope and appropriate use of the term probiotic. Nat Rev Gastroenterol Hepatol (2014) 11:506–14. doi: 10.1038/nrgastro.2014.66 24912386

[114] Douillard FP de Vos WM. Functional genomics of lactic acid bacteria: from food to health. Microb Cell Fact (2014) 13:S8. doi: 10.1186/1475-2859-13-S1-S8 25186768 PMC4155825

[115] Lin T-L Shu C-C Lai W-F Tzeng C-M Lai H-C Lu C-C. Investiture of next generation probiotics on amelioration of diseases – strains do matter. Med Microecol (2019) 1–2:100002. doi: 10.1016/j.medmic.2019.100002

[116] Zhang T Li Q Cheng L Buch H Zhang F. *Akkermansia muciniphila* is a promising probiotic. Microb Biotechnol (2019) 12:1109–25. doi: 10.1111/1751-7915.13410 PMC6801136 31006995

[117] Ouwerkerk JP Aalvink S Belzer C De Vos WM. Preparation and preservation of viable *Akkermansia muciniphila* cells for therapeutic interventions. Benef Microbes (2017) 8:163–9. doi: 10.3920/BM2016.0096 28116930

[118] de Almada CN Almada CN Martinez RCR Sant’Ana AS. Paraprobiotics: Evidences on their ability to modify biological responses, inactivation methods and perspectives on their application in foods. Trends Food Sci Technol (2016) 58:96–114. doi: 10.1016/j.tifs.2016.09.011

[119] Druart C Plovier H Van Hul M Brient A Phipps KR Vos WM. Toxicological safety evaluation of pasteurized Akkermansia muciniphila. J Appl Toxicol (2021) 41:276–90. doi: 10.1002/jat.4044 PMC7818173 32725676

[120] Ashrafian F Keshavarz Azizi Raftar S Shahryari A Behrouzi A Yaghoubfar R Lari A. Comparative effects of alive and pasteurized akkermansia muciniphila on normal diet-fed mice. Sci Rep (2021) 11:17898. doi: 10.1038/s41598-021-95738-5 34504116 PMC8429653

[121] Grajeda-Iglesias C Durand S Daillère R Iribarren K Lemaitre F Derosa L. Oral administration of akkermansia muciniphila elevates systemic antiaging and anticancer metabolites. Aging (2021) 13:6375–405. doi: 10.18632/aging.202739 PMC7993698 33653967

[122] Salminen S Collado MC Endo A Hill C Lebeer S Quigley EMM. The international scientific association of probiotics and prebiotics (ISAPP) consensus statement on the definition and scope of postbiotics. Nat Rev Gastroenterol Hepatol (2021) 18:649–67. doi: 10.1038/s41575-021-00440-6 PMC8387231 33948025

[123] Ghaderi F Sotoodehnejadnematalahi F Hajebrahimi Z Fateh A Siadat SD. Effects of active, inactive, and derivatives of akkermansia muciniphila on the expression of the endocannabinoid system and PPARs genes. Sci Rep (2022) 12:10031. doi: 10.1038/s41598-022-13840-8 35705595 PMC9200819

[124] Yaghoubfar R Behrouzi A Zare Banadkoki E Ashrafian F Lari A Vaziri F. Effect of akkermansia muciniphila, faecalibacterium prausnitzii, and their extracellular vesicles on the serotonin system in intestinal epithelial cells. Probiotics Antimicrob Proteins (2021) 13:1546–56. doi: 10.1007/s12602-021-09786-4 33852147

[125] Cani PD Possemiers S Van de Wiele T Guiot Y Everard A Rottier O. Changes in gut microbiota control inflammation in obese mice through a mechanism involving GLP-2-driven improvement of gut permeability. Gut (2009) 58:1091–103. doi: 10.1136/gut.2008.165886 PMC2702831 19240062

[126] Shin N-R Lee J-C Lee H-Y Kim M-S Whon TW Lee M-S. An increase in the *Akkermansia* spp. population induced by metformin treatment improves glucose homeostasis in diet-induced obese mice. Gut (2014) 63:727–35. doi: 10.1136/gutjnl-2012-303839 23804561

[127] Katiraei S Vries MR Costain AH Thiem K Hoving LR Diepen JA. *Akkermansia muciniphila* exerts lipid-lowering and immunomodulatory effects without affecting neointima formation in hyperlipidemic APOE*3-Leiden.CETP mice. Mol Nutr Food Res (2020) 64:1900732. doi: 10.1002/mnfr.201900732 31389129 PMC7507188

[128] Depommier C Van Hul M Everard A Delzenne NM De Vos WM Cani PD. Pasteurized *Akkermansia muciniphila* increases whole-body energy expenditure and fecal energy excretion in diet-induced obese mice. Gut Microbes (2020) 11:1231–45. doi: 10.1080/19490976.2020.1737307 PMC7524283 32167023

[129] Luo Z-W Xia K Liu Y-W Liu J-H Rao S-S Hu X-K. Extracellular vesicles from akkermansia muciniphila elicit antitumor immunity against prostate cancer via modulation of CD8+ T cells and macrophages. IJN (2021) 16:2949–63. doi: 10.2147/IJN.S304515 PMC8068512 33907401

[130] Meng X Wang W Lan T Yang W Yu D Fang X. A purified aspartic protease from akkermansia muciniphila plays an important role in degrading Muc2. IJMS (2019) 21:72. doi: 10.3390/ijms21010072 31861919 PMC6982040

[131] Meng X Zhang J Wu H Yu D Fang X. Akkermansia muciniphila aspartic protease Amuc_1434* inhibits human colorectal cancer LS174T cell viability *via* TRAIL-mediated apoptosis pathway. IJMS (2020) 21:3385. doi: 10.3390/ijms21093385 32403433 PMC7246985

[132] Wang F Cai K Xiao Q He L Xie L Liu Z. *Akkermansia muciniphila* administration exacerbated the development of colitis-associated colorectal cancer in mice. J Cancer (2022) 13:124–33. doi: 10.7150/jca.63578 PMC8692691 34976176

[133] Keshavarz Azizi Raftar S Abdollahiyan S Azimirad M Yadegar A Vaziri F Moshiri A. The anti-fibrotic effects of heat-killed akkermansia muciniphila MucT on liver fibrosis markers and activation of hepatic stellate cells. Probiotics Antimicro Prot (2021) 13:776–87. doi: 10.1007/s12602-020-09733-9 33433897

[134] Keshavarz Azizi Raftar S Ashrafian F Yadegar A Lari A Moradi HR Shahriary A. The protective effects of live and pasteurized akkermansia muciniphila and its extracellular vesicles against HFD/CCl4-induced liver injury. Microbiol Spectr (2021) 9:e00484–21. doi: 10.1128/Spectrum.00484-21 PMC8557882 34549998

[135] Raftar SKA Ashrafian F Abdollahiyan S Yadegar A Moradi HR Masoumi M. The anti-inflammatory effects of akkermansia muciniphila and its derivates in HFD/CCL4-induced murine model of liver injury. Sci Rep (2022) 12:2453. doi: 10.1038/s41598-022-06414-1 35165344 PMC8844054

[136] Rao Y Kuang Z Li C Guo S Xu Y Zhao D. Gut akkermansia muciniphila ameliorates metabolic dysfunction-associated fatty liver disease by regulating the metabolism of l-aspartate *via* gut-liver axis. Gut Microbes (2021) 13:1927633. doi: 10.1080/19490976.2021.1927633 34030573 PMC8158032

[137] Wu F Guo X Zhang M Ou Z Wu D Deng L. An akkermansia muciniphila subtype alleviates high-fat diet-induced metabolic disorders and inhibits the neurodegenerative process in mice. Anaerobe (2020) 61:102138. doi: 10.1016/j.anaerobe.2019.102138 31830598

[138] Yang M Bose S Lim S Seo J Shin J Lee D. Beneficial effects of newly isolated akkermansia muciniphila strains from the human gut on obesity and metabolic dysregulation. Microorganisms (2020) 8:1413. doi: 10.3390/microorganisms8091413 32937828 PMC7564497

[139] Ashrafian F Keshavarz Azizi Raftar S Lari A Shahryari A Abdollahiyan S Moradi HR. Extracellular vesicles and pasteurized cells derived from akkermansia muciniphila protect against high-fat induced obesity in mice. Microb Cell Fact (2021) 20:219. doi: 10.1186/s12934-021-01709-w 34863163 PMC8645101

[140] Shi M Yue Y Ma C Dong L Chen F. Pasteurized akkermansia muciniphila ameliorate the LPS-induced intestinal barrier dysfunction *via* modulating AMPK and NF-κB through TLR2 in caco-2 cells. Nutrients (2022) 14:764. doi: 10.3390/nu14040764 35215413 PMC8879293

[141] Yoon HS Cho CH Yun MS Jang SJ You HJ Kim J. Akkermansia muciniphila secretes a glucagon-like peptide-1-inducing protein that improves glucose homeostasis and ameliorates metabolic disease in mice. Nat Microbiol (2021) 6:563–73. doi: 10.1038/s41564-021-00880-5 33820962

[142] Ashrafian F Shahriary A Behrouzi A Moradi HR Keshavarz Azizi Raftar S Lari A. Akkermansia muciniphila-derived extracellular vesicles as a mucosal delivery vector for amelioration of obesity in mice. Front Microbiol (2019) 10:2155. doi: 10.3389/fmicb.2019.02155 31632356 PMC6779730

[143] Hänninen A Toivonen R Pöysti S Belzer C Plovier H Ouwerkerk JP. *Akkermansia muciniphila* induces gut microbiota remodelling and controls islet autoimmunity in NOD mice. Gut (2018) 67:1445–53. doi: 10.1136/gutjnl-2017-314508 29269438

[144] Chelakkot C Choi Y Kim D-K Park HT Ghim J Kwon Y. Akkermansia muciniphila-derived extracellular vesicles influence gut permeability through the regulation of tight junctions. Exp Mol Med (2018) 50:e450–0. doi: 10.1038/emm.2017.282 PMC5903829 29472701

[145] Ke H Li F Deng W Li Z Wang S Lv P. Metformin exerts anti-inflammatory and mucus barrier protective effects by enriching akkermansia muciniphila in mice with ulcerative colitis. Front Pharmacol (2021) 12:726707. doi: 10.3389/fphar.2021.726707 34658866 PMC8514724

[146] Kang C Ban M Choi E-J Moon H-G Jeon J-S Kim D-K. Extracellular vesicles derived from gut microbiota, especially akkermansia muciniphila, protect the progression of dextran sulfate sodium-induced colitis. PloS One (2013) 8:e76520. doi: 10.1371/journal.pone.0076520 24204633 PMC3811976

[147] Qian K Chen S Wang J Sheng K Wang Y Zhang M. A β- *N* -acetylhexosaminidase Amuc_2109 from *Akkermansia muciniphila* protects against dextran sulfate sodium-induced colitis in mice by enhancing intestinal barrier and modulating gut microbiota. Food Funct (2022) 13:2216–27. doi: 10.1039/D1FO04094D 35133390

[148] Wang J Xu W Wang R Cheng R Tang Z Zhang M. The outer membrane protein Amuc_1100 of *Akkermansia muciniphila* promotes intestinal 5-HT biosynthesis and extracellular availability through TLR2 signalling. Food Funct (2021) 12:3597–610. doi: 10.1039/D1FO00115A 33900345

[149] Hu X Zhao Y Yang Y Gong W Sun X Yang L. Akkermansia muciniphila improves host defense against influenza virus infection. Front Microbiol (2021) 11:586476. doi: 10.3389/fmicb.2020.586476 33603716 PMC7884316

[150] Mulhall H DiChiara JM Huck O Amar S. Pasteurized *Akkermansia muciniphila* reduces periodontal and systemic inflammation induced by *Porphyromonas gingivalis* in lean and obese mice. J Clin Periodontol (2022) 49:717–29. doi: 10.1111/jcpe.13629 35415929

[151] Lawenius L Scheffler JM Gustafsson KL Henning P Nilsson KH Colldén H. Pasteurized *Akkermansia muciniphila* protects from fat mass gain but not from bone loss. Am J Physiology-Endocrinol Metab (2020) 318:E480–91. doi: 10.1152/ajpendo.00425.2019 PMC7191407 31961709

[152] Yaghoubfar R Behrouzi A Ashrafian F Shahryari A Moradi HR Choopani S. Modulation of serotonin signaling/metabolism by akkermansia muciniphila and its extracellular vesicles through the gut-brain axis in mice. Sci Rep (2020) 10:22119. doi: 10.1038/s41598-020-79171-8 33335202 PMC7747642

[153] Ding Y Bu F Chen T Shi G Yuan X Feng Z. A next-generation probiotic: Akkermansia muciniphila ameliorates chronic stress–induced depressive-like behavior in mice by regulating gut microbiota and metabolites. Appl Microbiol Biotechnol (2021) 105:8411–26. doi: 10.1007/s00253-021-11622-2 34617139

[154] Depommier C Everard A Druart C Maiter D Thissen J-P Loumaye A. Serum metabolite profiling yields insights into health promoting effect of a. muciniphila in human volunteers with a metabolic syndrome. Gut Microbes (2021) 13:1994270. doi: 10.1080/19490976.2021.1994270 34812127 PMC8632301

[155] Finegold SM Li Z Summanen PH Downes J Thames G Corbett K. Xylooligosaccharide increases bifidobacteria but not lactobacilli in human gut microbiota. Food Funct (2014) 5:436. doi: 10.1039/c3fo60348b 24513849

[156] Maier TV Lucio M Lee LH VerBerkmoes NC Brislawn CJ Bernhardt J. Impact of dietary resistant starch on the human gut microbiome, metaproteome, and metabolome. mBio (2017) 8:e01343-17. doi: 10.1128/mBio.01343-17 29042495 PMC5646248

[157] Remely M Hippe B Geretschlaeger I Stegmayer S Hoefinger I Haslberger A. Increased gut microbiota diversity and abundance of faecalibacterium prausnitzii and akkermansia after fasting: A pilot study. Wien Klin Wochenschr (2015) 127:394–8. doi: 10.1007/s00508-015-0755-1 PMC4452615 25763563

[158] Gibson GR Roberfroid MB. Dietary modulation of the human colonic microbiota: Introducing the concept of prebiotics. J Nutr (1995) 125:1401–12. doi: 10.1093/jn/125.6.1401 7782892

[159] Lordan C Thapa D Ross RP Cotter PD. Potential for enriching next-generation health-promoting gut bacteria through prebiotics and other dietary components. Gut Microbes (2020) 11:1–20. doi: 10.1080/19490976.2019.1613124 31116628 PMC6973326

[160] Anhê FF Roy D Pilon G Dudonné S Matamoros S Varin TV. A polyphenol-rich cranberry extract protects from diet-induced obesity, insulin resistance and intestinal inflammation in association with increased *Akkermansia* spp. popul gut microbiota mice Gut (2015) 64:872–83. doi: 10.1136/gutjnl-2014-307142 25080446

[161] Roopchand DE Carmody RN Kuhn P Moskal K Rojas-Silva P Turnbaugh PJ. Dietary polyphenols promote growth of the gut bacterium *Akkermansia muciniphila* and attenuate high-fat diet–induced metabolic syndrome. Diabetes (2015) 64:2847–58. doi: 10.2337/db14-1916 PMC4512228 25845659

[162] Zhang L Carmody RN Kalariya HM Duran RM Moskal K Poulev A. Grape proanthocyanidin-induced intestinal bloom of akkermansia muciniphila is dependent on its baseline abundance and precedes activation of host genes related to metabolic health. J Nutr Biochem (2018) 56:142–51. doi: 10.1016/j.jnutbio.2018.02.009 PMC5971143 29571008

[163] Masumoto S Terao A Yamamoto Y Mukai T Miura T Shoji T. Non-absorbable apple procyanidins prevent obesity associated with gut microbial and metabolomic changes. Sci Rep (2016) 6:31208. doi: 10.1038/srep31208 27506289 PMC4979010

[164] Abot A Brochot A Pomié N Wemelle E Druart C Régnier M. Camu-camu reduces obesity and improves diabetic profiles of obese and diabetic mice: A dose-ranging study. Metabolites (2022) 12:301. doi: 10.3390/metabo12040301 35448490 PMC9025096

[165] Shang Q Song G Zhang M Shi J Xu C Hao J. Dietary fucoidan improves metabolic syndrome in association with increased akkermansia population in the gut microbiota of high-fat diet-fed mice. J Funct Foods (2017) 28:138–46. doi: 10.1016/j.jff.2016.11.002

[166] Everard A Lazarevic V Derrien M Girard M Muccioli GG Neyrinck AM. Responses of gut microbiota and glucose and lipid metabolism to prebiotics in genetic obese and diet-induced leptin-resistant mice. Diabetes (2011) 60:2775–86. doi: 10.2337/db11-0227 PMC3198091 21933985

[167] Neyrinck AM Sánchez CR Rodriguez J Cani PD Bindels LB Delzenne NM. Prebiotic effect of berberine and curcumin is associated with the improvement of obesity in mice. Nutrients (2021) 13:1436. doi: 10.3390/nu13051436 33923174 PMC8145536

[168] Bu F Ding Y Chen T Wang Q Wang R Zhou J-Y. Total flavone of abelmoschus manihot improves colitis by promoting the growth of akkermansia in mice. Sci Rep (2021) 11:20787. doi: 10.1038/s41598-021-00070-7 34675239 PMC8531128

[169] Régnier M Rastelli M Morissette A Suriano F Le Roy T Pilon G. Rhubarb supplementation prevents diet-induced obesity and diabetes in association with increased akkermansia muciniphila in mice. Nutrients (2020) 12:2932. doi: 10.3390/nu12102932 32987923 PMC7601677

[170] Payahoo L Khajebishak Y Alivand MR Soleimanzade H Alipour S Barzegari A. Investigation the effect of oleoylethanolamide supplementation on the abundance of akkermansia muciniphila bacterium and the dietary intakes in people with obesity: A randomized clinical trial. Appetite (2019) 141:104301. doi: 10.1016/j.appet.2019.05.032 31132422

