# Supplementary material for: Akkermansia muciniphila: The state of the art, 18 years after its first discovery
Source: Front Gastroenterol (Lausanne). 2022 Oct 25;1:1024393. doi: 10.3389/fgstr.2022.1024393 (PMC12952328; doi:10.3389/fgstr.2022.1024393)
Supplement: Supplementary file 1 [file DataSheet_1.docx]

Supplementary Data

# Methodology

A rigorous literature search was performed to select studies published between 2004 and March 2022. The search engines used were PubMed, Web of Science, Google Scholar and Scopus. The keywords used in the searches included “*Akkermansia muciniphila*” and “genomic characteristics”, “phenotypic characteristics”, “culture”, “oral microbiota”, “gut microbiota”, “urinary microbiota”, “health”, “cancer”, “metabolic disorders”, “diseases”, and “probiotic”. Additional useful records referenced in the selected studies were ultimately added. All duplicate studies were removed. The articles were then screened based on the titles and abstracts. Finally, the full texts of the remaining studies were screened, and those which met the criteria and the subjects being discussed were included in this review.

The inclusion criteria of the involved studies were as follows:

(1) studies analyzing the genomic and phenotypical characteristics of *A. muciniphila*;

(2) studies that provide information about its distribution and abundance in the different human microbiota;

(3) studies that provided information about its relationship and the variation in its abundance in pathological situations;

(4) studies about the potential role of *A.muciniphila* as a probiotic.

Review articles, meta-analysis articles and articles which were unavailable were excluded.

Two authors (RI and RM) screened the studies and extracted information for each eligible paper. The data extracted included the following variables: the first author’s name, publication year, sample type, cohort, different methods used in the study and, finally, the main findings or results of each study. A total of 536 potentially relevant articles were identified in the initial electronic search, including 210 from PubMed, 95 from Web of Science, 53 from Scopus, and 178 from Google Scholar. Duplicates (n =82) were removed, and the titles and/or abstracts of the 454 remaining studies were screened for relevance. Of these 454 studies, 128 were selected and included in this review, and 30 other articles were added after screening the selected studies and their references (figure 1).
